# The role of biotics and bioactive compounds in sports injuries: a narrative review

**DOI:** 10.3389/fnut.2026.1813030

**Published:** 2026-05-14

**Authors:** Kezban Şahin-Demirci, Nursel Dal, Buket Gönen-Çolak, Onur Akman, Bence Raposa, Duygu Ağagündüz

**Affiliations:** 1 Department of Nutrition and Dietetics, Faculty of Health Sciences, Bandirma Onyedi Eylul University, Balıkesir, Türkiye; 2 Department of Coaching Education, Faculty of Sports Sciences, Bayburt University, Bayburt, Türkiye; 3 Faculty of Health Sciences, Institute of Basics of Health Sciences, Midwifery and Health Visiting, University of Pécs, Pécs, Hungary; 4 Department of Nutrition and Dietetics, Faculty of Health Sciences, Gazi University, Ankara, Türkiye

**Keywords:** polyphenols, probiotics, sports injuries, sports nutrition, supplementation

## Abstract

Sports injuries affect not just the physical, but also the psychological, social, and professional aspects of athletes’ lives. Preventive strategies, nutrition, and nutritional supplements—especially biotics and bioactive compounds—are crucial for recovery and may help prevent injury recurrence. This review examines biotics and bioactive compounds that may be effective on sports injuries, revealing their potential benefits and safety of use. Using a comparative and interpretive approach, the pertinent literature was thoroughly examined for this review, with a special focus on clinical and preclinical research conducted within the last 5 years. Bioactive compounds and biotics (prebiotics: indigestible substances that support beneficial bacteria; probiotics: live beneficial microorganisms; postbiotics: bioactive microbial metabolites) can improve sports-related injuries by affecting inflammation, mitochondrial function, biogenesis, oxidative stress, and atrophy. Biotics achieve these systemic effects by improving intestinal barrier function and modulating the microbiota. Current literature indicates that some compounds show promise for treating sports injuries, but human study evidence is limited. Animal models provide useful insights for future research. Finally, polyphenolic compounds, amino acids/peptides, and w-3 fatty acids, thanks to their anti-inflammatory and antioxidant properties, can indirectly contribute to muscle regeneration and cell repair, thus supporting the recovery process in athletes. However, it is still too early to make recommendations regarding the use of biotics and bioactive compounds in sports injuries.

## 1 Introduction

With the promotion of healthy living and the increased interest in physical activity today, the risk of injury and trauma has also increased (1). Physical activity-related injuries or exercise-induced muscle damage are experienced by all individuals, from athletes to sedentary individuals (2). Sports injuries generally affect the musculoskeletal system, and muscles, bones, joints, ligaments, and tendons can be damaged (3). Mechanical stress and metabolic processes within the musculoskeletal system interact to produce both sports injuries and exercise-induced muscle damage. Exercise-induced muscle injury typically involves microscopic structural disruption of muscle fibers, particularly during eccentric contractions, while sports injuries generally result from acute trauma or repetitive strain. In this process, proteolytic enzymes become activated, and an inflammatory response arises due to sarcomere injury and disruption of calcium homeostasis (2–5). Although named differently depending on the location and tissue structure of the injury, they can lead to immobility, causing rapid loss of strength, muscle atrophy, and impaired training adaptation over time (6). This creates significant concern not only for individual athletes and sports federations but also for healthcare services (7, 8).

It is noted that sports injuries frequently recur, and subsequent injuries of the similar or various types can be significantly impacted by prior injuries (9). Therefore, preventing sports injuries is crucial, both to reduce the economic costs incurred in rehabilitation and surgical procedures after sports injuries and to improve the health benefits of athletes. Sport-specific programs should be developed, taking into account injury patterns and risk factors (10). Regarding this, nutrition and nutritional supplements are crucial for promoting return to exercise, speeding up recuperation, and lessening the detrimental consequences of inactivity. Beginning in the early phases of the healing process, nutritional intervention is essential for supplying the energy and nutrients required to promote tissue regeneration and wound healing as well as to manage oxidative stress and inflammation. Athletes’ anabolism can be greatly aided by high-quality proteins, lipids, vitamins, antioxidants, minerals, and other supplements. Therefore, adequate and balanced nutrition is critical for injury prevention and recovery. However, supplementation of certain compounds that cannot be obtained through diet may also be necessary for these processes (11–15). Although limited to a small number of clinical studies so far, it has been reported that probiotics and postbiotics support the healing process through the control of oxidative stress, reduction of inflammation, and immune regulation (16–19), and that symbiotics and prebiotics support endurance and muscle recovery by increasing short-chain fatty acid production (20, 21).

It has been suggested that non-nutritive but bioactive compounds that can positively affect health, such as polyphenols, carotenoids, omega-3 fatty acids, phytochemicals, and dietary fiber, may also be promising for sports injuries. Oxidative stress can be prevented by carotenoids, polyphenols, flavonoids, micronutrients like vitamins E and C, and antioxidant minerals; the anti-inflammatory and antioxidant properties of omega-3 fatty acids and curcumin can benefit the rehabilitation process (13–15). Particularly, the potential of adequate intake of nutrients such as creatine, curcumin, omega-3 fatty acids, and antioxidants to help with wound healing during the period of immobilization phase after injury is emphasized (22). Furthermore, optimal vitamin D and calcium levels support bone healing, while leucine and creatine can play important roles in protein synthesis and muscle damage repair (13, 23, 24). However, while deficiencies in these nutrients slow healing, evidence that supplementation accelerates healing is quite limited (11, 15, 22). Furthermore, the role of nutrients and non-nutritives in athlete rehabilitation is a field with high potential but is not yet fully developed, especially considering the time lost due to injuries (13, 15). Therefore, strong evidence is needed for the role of biotics and bioactive compounds in optimizing the prevention and clinical management of sports injuries. Accordingly, this review thoroughly examines biotics and bioactive compounds that may be effective on sports injuries, presenting their potential benefits and safety of use. The review looked at all the relevant literature in detail, focusing on clinical and preclinical studies from the last five years and using both a comparative and an interpretive method.

## 2 An overview of sports injuries

Impairments affecting musculoskeletal tissues, such as bones, joints, muscles, tendons, ligaments, fascia, and synovial structures, with the potential impact of nearby vascular and neurological components, are referred to as sports injuries. Acute sports-related trauma or cumulative overuse linked to repetitive mechanical loading are frequently blamed for these injuries, which show up as clinical symptoms and signs (25, 26). Sports injuries are especially usual in high-performance environments and can negatively impact athletic availability and performance. They can also be linked to psychological stress and, in certain situations, lead to early career termination (6).

**FIGURE 1:**
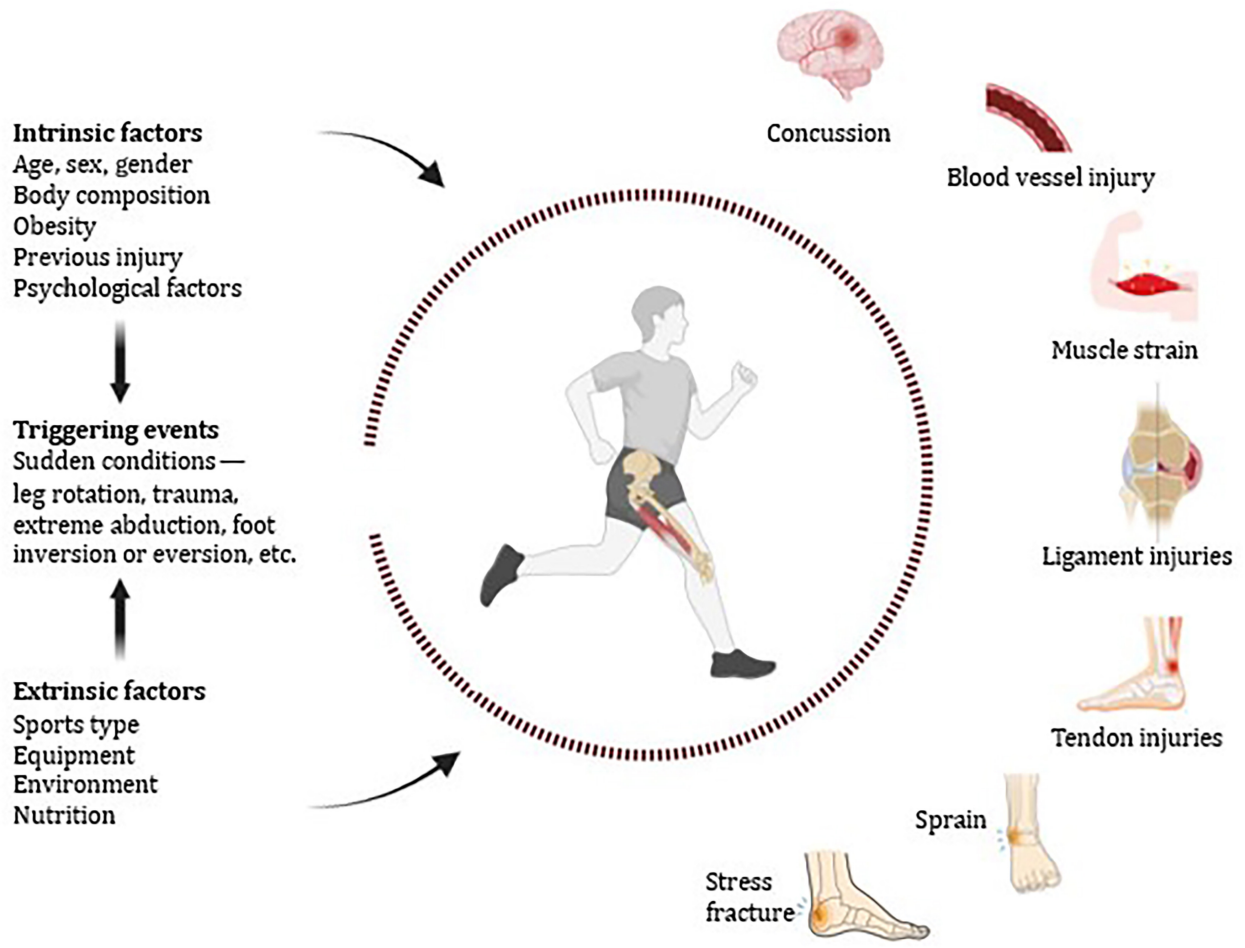
Factors and outcomes of sports injuries.

It seems that mechanical and biological factors interact to increase the risk of sports injuries. Intrinsic traits (e.g., age, sex/gender, and body composition), external factors (e.g., equipment and environmental conditions), and inciting movement-related events (e.g., leg rotation or forced abduction) that may cause tissue failure are frequently mentioned contributors (27). While ligament injuries are usually characterized by disruption of collagen fiber structure under injurious loading conditions, tendon injuries can result from direct trauma or excessive strain loading (28) (Figure 1). Depending on the severity of the injury, the management pathway may include a period of rest or immobilization. Meanwhile, tissue repair is progressing through the overlapping inflammatory, proliferative, and remodeling stages, and immobilization may lead to muscle atrophy, including loss of muscle size and function. Rehabilitation afterwards normally consists of a gradual increase in physical activity and loading, the regained limb is often the focus of such efforts to restore sporting activities (11). In the long run, sports injuries can lead to decreased strength, muscle wasting and less effective training adaptation (6), which in turn can affect both physical and mental health as well as overall quality of life (QoL) (2).

Injury patterns depend on the type of sport and the population. It is often said that injuries occur more frequently in contact sports, and differences in injury severity have been noted across various sports (29). Injuries have been reported to account for 45% of incidents among Japanese collegiate athletes and have been linked to considerable time lost from both training and competition (30). In Spain, a study on the surveillance of injuries in adolescent athletes revealed that 40.4% of the athletes had suffered from at least one injury during the period under review (31). A meta-analysis (*n* = 25.683) of athletes with disabilities showed that the overall injury rate was 30.9%, with acute traumatic and upper extremity injuries being commonly reported (32).

### 2.1 Types of sports injuries

Sports injuries typically get diagnosed by combining the athlete’s medical history (including the mechanism of the injury and pertinent background), physical examination, and, if clinically necessary, laboratory and imaging results. Bone and cartilage injuries, neurovascular injuries involving peripheral nerves and/or blood vessels, and soft-tissue injuries (such as muscle, tendon, fascia, ligament, and bursal injuries) can all be categorized based on the predominant tissue involved (8). Depending on the pattern of onset and duration of symptoms, injuries are also frequently classified as either acute or chronic based on their clinical time course. Non-osseous musculoskeletal structures, such as muscles, tendons, fascia, ligaments, and bursae, are the main targets of soft-tissue injuries; however, depending on the mechanism and severity of the injury, adjacent cartilage or neurovascular structures may also be affected (33) (Table 1).

**TABLE 1:** Common types of sports injuries.

Time-based sports injuries	
Acute injuries	Sprain (ankle), muscle strain, contusion, fracture, dislocation
Chronic or overuse injuries	Tendinitis, stress fracture, bursitis, chronic muscle, or tendon strain
**Tissue-based sports injuries**	
Muscle–tendon injuries	Muscle tear, tendon rupture, tendinitis
Ligament injuries	Sprain, anterior cruciate ligament (ACL), collateral ligament injury
Bone and cartilage injuries	Fractures, stress fractures
Joint injuries	Dislocation, meniscus injury
Soft-tissue injuries	Skin, fascia, bursitis, and connective tissue damage
Nerve/vascular injury	Trauma, peripheral nerve compression, laceration, arterial injury

Particular sports movement demands and loading patterns are linked to unique injury profiles which affect specific tissues and anatomical regions (e.g., lateral elbow pain syndromes usually referred to as “tennis elbow” and knee pain syndromes commonly referred to as “runner’s knee”) (27). Ankle, hamstring, and anterior cruciate ligament injuries are frequently reported in football players (10). Knee and foot/ankle injuries are common in volleyball and basketball, and handball players also have reported knee injuries (34). Muscular injuries are frequently reported among elite athletes, with potential negative effects on training continuity and performance (12), and bone stress injuries (including stress fractures) and tendinopathies represent significant clinical issues encountered in athletic populations (13). Direct trauma and/or high-load lengthening actions (often involving eccentric contractions) can cause muscle injuries, which can impair muscle function, reduce range of motion, and contribute to strength deficits. The degree and duration of impairment depend on the severity of the injury and the recovery processes (35). Acute muscle injury can cause structural disruption and necrosis of the afflicted muscle fibers at the tissue level, which sets off subsequent inflammatory and repair reactions (28).

### 2.2 Risk factors

As shown in Figure 1, intrinsic and extrinsic several factors can contribute to sports injuries, including training intensity, inadequate warm-up, and overuse of equipment or a specific area, depending on the sport (29, 30, 36, 37). Prolonged, intense exercise and strenuous training have been linked to a higher risk of injury in athletes (6). Age, gender, overweight or obesity, training habits, inadequate recovery due to early return to sport, and movement abnormalities are associated with injury and repetitive injury (30, 38–40).

It has been noted that adults suffer fewer injuries in sports compared to children whose reflexes and coordination are not yet fully developed (29). With aging, the decrease in bone density and the flexibility of muscles, ligaments, and tendons makes the body more vulnerable, increasing susceptibility to injury (27). According to a systematic review, hormonal, anatomical, and training variations contribute to a higher injury risk among female athletes compared to males (41). Kimura et al. reported that moderate to severe injuries (upper extremity, neck, and head injuries) are more common in males than in females, while milder and minimal injuries, particularly those affecting the lower extremities, are more frequently observed in female athletes (30).

The type of sport is the first determining factor to consider for injury risk (42). Although football is the most widely played sport worldwide, it is associated with a greater risk of injury than many other sports (39). The highest rates of sports injuries were observed in football (42.2%), running (36.3%), and cycling (21.5%), with 36.3% of athletes experiencing repetitive injuries (40). An investigation of competition-associated injuries in Olympic combat sports revealed that athletes competing in boxing, judo, and taekwondo experienced a significantly higher risk of injury than those participating in wrestling (43). Moreover, similar mechanisms, risk factors, or limited tissue healing from a previous injury can lead to recurrent sports injuries (9, 30). Conversely, interpersonal and sociocultural factors are more strongly linked to the development of overuse injuries, while stress-related reactions are the main psychosocial risk factors for acute injuries (37).

Nutrition and adequate fluid intake are critical for supporting performance, preventing injuries, and accelerating recovery in athletes (14). Inadequate or unbalanced nutrition can increase the risk of sports injuries and delay post-injury recovery and rehabilitation (13). Particularly, Low Energy Availability (LEA; <30 kcal/kg FFM/day), which refers to insufficient amounts of many nutrients, including calcium, iron, vitamin D, carbs, and protein, as well as energy intake in relation to exercise energy expenditure, has been associated with sports injuries (44). Insufficient dietary intake of energy and macronutrients increases the risk of injuries to muscles, tendons, and ligaments. Inadequate micronutrient intake adversely affects bone and muscle health. Additionally, dehydration and electrolyte imbalances elevate the risk of muscle cramps and exercise-induced muscle damage (6, 14, 45, 46). Therefore, to reduce the risk of sports injury, adequate intake of dietary energy, macronutrients, and micronutrients is crucial. Additionally, dietary bioactive compounds like polyphenols, carotenoids, and omega-3 fatty acids may offer extra defense against oxidative stress and inflammation brought on by exercise, potentially lowering the risk of injury and promoting tissue repair (35, 45). Moreover, biotics may modulate gut microbiota, reduce systemic inflammation, and enhance tissue repair during exercise, which could decrease injury risk and facilitate athlete recovery (20, 21). Thus, lowering the risk of injury in athletes necessitates an integrated dietary approach that includes micronutrients, bioactive compounds, and biotics in addition to sufficient energy and macronutrient intake.

### 2.3 Prevention

Sports injuries are a significant issue, as they cause detrimental long-term impacts on physical and mental health, negatively impacting both the performance and wellbeing of athletes (2). It is critical for everyone involved in sports to be aware of hydration, nutrition, team monitoring, and technical aspects to prevent injuries (29). Although sports injuries are often considered an inevitable consequence of sports, like other traumas and injuries, they can be prevented through both physical and other preventative approaches (10).

The proper intervention of evidence-based clinical nutrition is crucial for controlling immunological and metabolic health in sports injuries (6). A diet that provides adequate and balanced can help reduce inflammation during the healing process and minimize muscle mass loss during periods of immobilization (15, 47). Besides, adequate protein intake (1.5–2.0 g/kg/day) and certain micronutrients, such as vitamin C and zinc in the diet, can support injury healing (15). Regarding this, nutrition and appropriate nutritional supplements are important for accelerating recovery, enhancing immune function, and improving performance. The potential of various nutrients and functional substances to reduce oxidative stress and inflammation and enhance muscle strength recovery has been evaluated (11, 15, 22, 48, 49). Particularly, biotics and bioactive compounds such as polyphenols, omega-3 fatty acids, curcumin, and antioxidants may support healing in sports injuries and offer potential benefits in injury prevention (13, 17, 21). Recent studies on the dosage, duration, and effectiveness of these compounds are summarized in Tables 1–5.

**TABLE 2:** Some studies on the mechanisms of action of probiotics, prebiotics, and postbiotics in sports-related injuries.

Species/Strain	Dosage	Duration	Sports injuries	Animal/human characteristics	Potential mechanism of action	References
Probiotics						
Preclinical evidence						
*Lacticaseibacillus rhamnosus* IDCC3201	1 × 10^8^ CFU/day	4 weeks	Muscle atrophy	C57BL/6J mice	Decreased Atrogin-1 and MuRF1 expression; increased muscle mass and strength	(54)
*Lactiplantibacillus plantarum* strain WJL	1.8 × 10^9^ CFU/mL	6 weeks	Muscle and bone loss induced mice model	C57BL/6 mice	Increased IGF-1 expression; decreased Atrogin-1 and MuRF1 expression; increased muscle strength and endurance	(69)
*Lacticaseibacillus casei* Shirota	1 × 10^8^ or 1 × 10^9^ CFU/day	12 weeks	Aged mice model	SMAP8 mice	Increase in short-chain fatty acids (acetic, isobutyric, butyric, propionic, and hexanoic acids); decrease in age-related inflammation and reactive oxygen species increases	(62)
*Bifidobacterium animalis* subsp. lactis A6	2.0 × 10^10^ CFU/mL	4 weeks	DSS-induced mice	C57BL/6J mice	Inhibition of the NF-κB signaling pathway	(55)
*Lactiplantibacillus plantarum* HY7715	1 × 10^9^ CFU/kg/gün	5 weeks	Aged mice model	Balb/c male mice	Decreased Atrogin-1 and MuRF1 expression; decreased TNF-α levels	(61)
*Limosilactobacillus fermentum*	10^9^ CFU/day	8 and 12 weeks	Muscle atrophy	Female Balb/c mice	Decreased IL-6, TNF-α, and Hsp60 expression; increased MyoD expression in satellite cells	(210)
*Lacticaseibacillus casei* fermented milk	1.5 × 10^8^ CFU/mL	28 days	Mouse model with decreased bone mineral density after antibiotic treatment	Female Kunming mice	Decrease in RANKL and Ang II levels; increase in α smooth muscle actin expression in callus tissue	(58)
*Bifidobacterium longum*	1 × 10^8^ and/or 1 × 10^9^ CFU	3, 7, 14, 21, or 35 days	Aged mice with induced bone fractures	C57BL/6JN mice	Acceleration of bone callus formation; decrease in IL-10 and serum lipocalin-2 levels	(211)
*Lacticaseibacillus rhamnosus* GG	1 × 10^9^ CFU/day	6 weeks	Ovariectomized rats	Sprague-Dawley rats	Decreased TNF-α and IL-17 expression; increased IL-10 and TGF-β expression; decreased RANKL levels; decreased Th17/Treg ratio	(212)
*Clostridium butyricum*	5 × 108 CFU	12 weeks	Radiation-induced bone loss mouse model	Female C57BL/6 J mice	Suppression of IL-17A, NF-κB signaling pathway, decrease in Th17/Treg ratio	(60)
*Lactobacillus plantarum GKD7 (live or heat-killed)*	100 mg/kg	6 weeks	Osteoarthritis (anterior cruciate ligament transection)	Sprague-Dawley rats	Reduction of IL-1β and TNF-α expression in synovial tissue, inhibition of MMP3 in cartilage	(63)
*Akkermansia muciniphila*	8 × 108 CFU	6 weeks	Bone fracture	Female C57BL/6 mice	Improvement in intestinal barrier; reduction in inflammation; increased H-type vessel formation	(66)
Clinical (human) evidence						
*Lactococcus lactis subsp. lactis LY-66 and/or Lactobacillus plantarum PL-02*	7.5 × 10^9^ CFU	6 weeks	–	Healthy individuals (*n* = 88)	Increased muscle mass, decreased IL-6 levels, reduced strength loss, accelerated recovery of Rate of Force Development (RFD) and Relative Peak Force (RPF)	(213)
*Lacticaseibacillus paracasei* PS23 + omega 3 + lösin	30 billion CFU/day	2 months	–	Elderly individuals (*n* = 60)	Decrease in CRP levels; increase in muscle function	(72)
*Lactobacillus acidophilus and Bifidobacterium animalis subsp. lactis*	2 × 10^10^ CFU	30 days	–	Male marathon runners (*n* = 27)	Preservation of CD8+ T cell population; immunomodulatory effect with reduced pro-inflammatory cytokine response in stimulated lymphocytes	(75)
*Probiyotik (Ultra-biotic 60™) and Saccharomyces boulardii*	60 × 10^9^ CFU/day + *S. boulardii* (250 mg)	17 weeks	–	Elite male rugby player (*n* = 19)	Muscle soreness and leg heaviness sensation scores were significantly lower with probiotic intake	(74)
*Streptococcus thermophilus FP4 and Bifidobacterium breve BR03*	1 × ^10^ CFU	21 days	–	Healthy male resistance trainers (*n* = 15)	Reduced performance decline after muscle injury, preservation of joint range of motion, and decreased IL-6 levels	(76)
**Prebiotics**						
Preclinical evidence						
Pea protein + inulin	7.5% (w/w) inulin, 14% (w/w) pea protein	16 weeks	–	Male Wistar rats	Decreased MuRF1 expression; increased PGC1-α expression	(78)
Rice bran	10% (w/w)	12 weeks	–	Female ICR mice	Increased expression of occludin and ZO-1; decreased expression of IL-6 and TNF-alpha; decreased expression of MuRF-1 and atrogin-1	(83)
High-fiber, high-fat, and high-sugar diet	Diet containing 6.93/100 g soluble fiber (approximately three times more than the control)	10 weeks	–	C57BL/6J mice	Increased levels of acetate, butyrate, and propionate; increased muscle endurance in female mice	(214)
Fiber-rich diet	5% Partially hydrolyzed guar gum	2 weeks	–	BALB/c mouse cachexia model	Decreased Atrogin-1 and MuRF1 expression; decreased IL-6 levels	(215)
Resveratrol + prebiotic fiber + omega-3 fatty acids	10% oligofructose	–	–	Sprague–Dawley rats	Relief of short-term memory deficits and depressive-like behaviors after traumatic brain injury	(82)
Inulin	%8 (w/w)	2 months	–	Male C57BL/6 wild-type mice	Alleviation of dysbiosis, preservation of fimbriae, inner and outer capsule white matter damage and cerebral blood flow in the right hippocampus following head trauma	(81)
Clinical (human) evidence						
Yeast beta-glucan (*Saccharomyces cerevisiae*)	250 mg/day	13 days	–	Active healthy individuals (*n* = 31)	Significant reduction in pro-inflammatory cytokines such as MIP-1β, IL-8 and MCP-1 after 72 hours; tendency to decrease in TNF-α	(84)
Inulin	20 g/day	6 weeks	–	Individuals with osteoarthritis (*n* = 117)	Decreased pain sensitivity; increased hand grip strength	(85)
**Postbiotics**						
Preclinical evidence						
Whey + *Lentilactobacillus kefir* DH5 + Melon rind polyphenol extract	10 mL/kg/day	3 weeks	Immobilization-induced muscle atrophy	C57BL/6J mice	Increased expression of Atrogin-1, IGF-1, and MyoD genes	(216)
Heat-killed *Lactiplantibacillus plantarum* strains + inulin from Cichorium intybus L. root	5,000 μg/kg/day and/or 10,000 μg/kg/day	19 days	DEX kaynaklı sarkopeni	DEX-induced sarcopenia	Decreased Atrogin-1 expression; suppression of p38 and ERK phosphorylation in the MAPK signaling pathway	(86)
*Lactobacillus kefiri* DH5 fermented, freeze-dried whey	10% (w/w)	5 weeks	Immobilization-induced muscle atrophy	C57BL/6J mice	Increased expression of Atrogin-1, IGF-1, and MyoD genes	(217)
Heat-killed *Lactiplantibacillus plantarum* beLP1	1 and/or 3 mg/kg/day	2 weeks	DEX-induced sarcopenia	Sprague–Dawley rats	Prevention of muscle mass loss; Decreased expression of Atrogin-1, MuRF1, and FoxO3α; Increased AKT phosphorylation	(90)
Live/heat-inactivated/fermented *Lacticaseibacillus casei* GCK1	57 mg/kg/day or 1,000 mg/kg/day	28 days	Postmenopausal osteoporosis model	Female ICR mice	Increase in bone mineral density; decrease in IL-17A levels	(218)
Clinical (human) evidence						
*Akkermansia muciniphila* HB05 pasteurized form (HB05P)	1 x 10^10^ cells/day	12 weeks	–	Individuals aged 60 and over	Increase in follistatin levels	(54)
Live and/or heat-treated *Lacticaseibacillus paracasei* PS23	2 × 10^10^ CFU/day	12 weeks	–	Individuals aged 65–85 (*n* = 100)	Decrease in CRP and IL-6 levels; increase in IL-10 levels; increase in lower extremity muscle strength and endurance	(73)
*Lacticaseibacillus paracasei PS23 (live or heat-killed)*	2 × 10^10^ CFU	6 weeks	–	Healthy young adults (*n* = 114)	Reduced muscle strength loss; decreased creatine kinase, myoglobin and hs-CRP levels	(92)
*Lactiplantibacillus plantarum TWK10*	3 × 10^10^ CFU	6 weeks	–	Healthy adult males (*n* = 30)	Increased hand grip strength; significantly increased muscle mass; lower serum lactate and ammonia levels during exercise and recovery periods	(91)

NF-κB, nuclear factor kappa-light-chain-enhancer of activated B cells; TNF-α, tumor necrosis factor alpha; IL-6, interleukin-6; DEX, dexamethasone; IL-17, interleukin-17; IL-10, interleukin-10; TGF-β, transforming growth factor beta; RANKL, receptor activator of nuclear factor kappa-B ligand; Th17, T helper 17 cells; Treg, regulatory T cells; CFU, colony forming unit; MIP-1β, macrophage inflammatory protein-1 beta; IL-8, interleukin-8; MCP-1, Monocyte Chemoattractant Protein-1.

**TABLE 3:** Some studies on the effects of polyphenols on sports injuries.

Species/strain	Dosage	Duration	Sports injuries	Animal/human characteristics	Potential mechanism of action	References
Curcumin turmeric	Maltodextrin placebo control group (0.1 g/kg/day) Group 1: intake of curcumin turmeric (1,000 mg/day) 1 h before the test Group 2: intake of curcumin turmeric (1,000 mg/day) immediately after the test	2 weeks	Muscle damage after resistance training	45 athletes from different active sports	Second-phase curcumin turmeric intake resulted in a significant decrease in CK and LDH levels compared to the first-phase and control groups.	(108)
Curcumin	Placebo Curcumin (270 mg/day)	2 weeks	Muscle damage	Six competitive female soccer players	↓ Serum levels of IL-6 − muscle damage indices	(110)
Resveratrol (RES)	Placebo (*n* = 12) RES-500 (500 mg RES/day, *n* = 12) RES-1000 (1,000 mg RES/day, *n* = 12)	7 days	Muscle damage following acute plyometric exercise	36 young males	↓ Serum levels of LDH and CK	(219)
Resveratrol	Normal control Mass-drop injury without any treatment (MDI) NSAID treatment (MDI + 10 mg/kg NSAID) RES supplementation (MDI + 25 mg/kg/day RES) RES supplementation and NSAID (MDI + resveratrol + NSAID)	7 days	Contusion-induced muscle injury	48-week-old male mice (*n* = 8 per group)	↓ Serum levels of uric acid, creatinine, LDH, and CK ↓ Muscle damage ↑ RES and RES + NSAID groups promoted muscle satellite cell regeneration	(113)
Quercetin	Placebo Quercetin (1 g/day)	14 days	Eccentric-induced muscle-damage	12 young male	↓ Serum levels of CK, LDH, and IL-6 ↑ Serum levels of IGF-I and IGF-II	(106)

RES, resveratrol; MDI, mass-drop injury, NSAID, non-steroidal anti-inflammatory drugs, CK, creatine kinase, LDH, lactate dehydrogenase; IL-6, interleukin-6; IGF-I, insulin-like growth factor I, IGF-II, insulin-like growth factor II.

**TABLE 4:** Animal and clinical studies on the mechanisms of action of ω-3 PUFAs in sports-related injuries.

Species/strain	Dosage	Duration	Sports injuries	Animal/human characteristics	Potential mechanism of action	References
w-3 PUFAs	Intraperitoneal, 2 mL/kg	30 min after TBI, once daily for three consecutive days	mTBI	C57BL/6 rats; Sham group (*n* = 15) TBI group (*n* = 15) Sham group + w-3 PUFA supplementation (*n* = 15)	NF-κB ↓, IL-1β↓, IL-6 ↓, and TNF-α↓ levels in the hippocampus via the PPARγ/NF-κB signaling pathway RIP1/β actin, RIP3/β actin, and MLKL/β actin levels ↓ Brain water content ↓ NSS ↓	(128)
w-3 PUFAs	Intraperitoneal, 2 mL/kg	30 min after TBI, once daily for 7 days	TBI	Sprague-Dawley rats; Sham group Sham group + w-3 PUFA supplementation After TBI, the subjects were divided into four subgroups: the 1-day group, the 3-day group, the 7-day group, and the 14-day group (each *n* = 12).	From day 3 onwards; NSS ↓ Brain water content ↓, improvement in brain edema, and enhanced blood-brain barrier permeability This was mediated by HMGB1/NF-κB pathway deacetylation via SIRT1 The regulation of microglial polarization. Starting from day 7; Improvement in rotarod performance	(129)
w-3 PUFAs	Intraperitoneal, 2 ml/kg)	30 min after TBI, once daily for 7 days	TBI	Sprague-Dawley rats; Sham group (*n* = 36) TBI group (*n* = 36) TBI + w-3 PUFA suplementation group (*n* = 36) After TBI, the subjects were divided into three subgroups: the 1-day group, the 3-day group, and the 7-day group.	3 days after injury; Upregulation of SIRT1 protein NF-κB p65 expression levels ↓ The expression of TLR4/NF-κB-related factors ↓ The expression of cleaved caspase-3 ↓ and Bax↓ in lesion areas of the cerebral cortex	(130)
w-3 PUFAs	3% Menhaden oil 1% Menhaden oil 10% Safflower oil	5 weeks	mTBI	Male C57BL/6 Mice Three groups: 1. High in n-3 PUFA (3N3) (*n* = 8) 2. Moderate n-3 PUFA (1N3) (1% Menhaden oil) (*n* = 8) 3. Control group: n-6 PUFA (0N3) (10% Safflower oil) (*n* = 8)	NSS ↓ Faster recovery after mTBI Neuroprotective protection	(131)
w-3 PUFAs	10% w/w safflower (Conversion of n-6 PUFA to n-3 PUFA)	9–10 weeks	TBI	Male *fat-1* mice (*n* = 12) (Male *fat-1* mice able to endogenously convert n-6 PUFA to n-3 PUFA) wild type (WT) mice (*n* = 12)	On the 7th day after WDI; NSS ↓ due to endogenous conversion in male fat-1 mice GFAP ↓ Neuronal damage ↓	(132)
w-3 PUFAs	Fish oil (4.6% EPA, 3.6% DHA) Oral gavage, 4.6 g/kg EPA and 3.8 g/kg DHA	Once a day for a duration of 1 month	Achilles tendon injury	Wistar rats; Sham group (*n* = 10): surgery without tendon damage Negative control (NC) group (*n* = 10): tendon damage Exercise group (*n* = 10): tendon damage w-3 supplement group (*n* = 10): tendon damage Exercise and w-3 supplement group (*n* = 10): tendon damage	In the exercise + w-3 group; TNF-α, IL-1β, and MDA levels ↓ Achilles tendon functionality index score ↓ MMP-3 and SCX gene expression ↑ MMP-9 gene expression ↓	(133)
DHA	2,4,6 g/day DHA	189 days	Head trauma	American-style football athletes (*n* = 81) 2 g/day DHA (*n* = 21) 4 g/day DHA (*n* = 19) 6 g/day DHA (*n* = 22) Placebo (*n* = 19)	DHA levels ↑ (dose-dependent) Nf-L levels ↓ in all three groups (dose-independent)	(123)
DHA + EPA	2.442 g/day DHA and 1.020 g/day EPA	7 months, five times a week	Sub-concussive head impacts	18–27 age group, male American football players *n* = 16 placebo; *n* = 11 DHA + EPA	Microstructural differences in any region of the brain → Differences in white matter measurement → The structural integrity of ascending white matter tracts is better preserved in the treatment group.	(122)
DHA + EPA	Fish oil (3.5 g/d DHA + EPA (2.4:1.0 ratio, ethyl ester form; 1 capsule: 407 mg/g DHA oil and 170 mg/g EPA) High-oleic safflower oil (1 capsule: 713 mg/g oleic acid oil and 130 mg/g linoleic acid oil)	26 weeks, 5 days a week	Repeated subconcussive head impacts	American football player Treatment group (*n* = 18) Placebo (*n* = 17)	NF-κB, IL-6, and TNF-α levels →	(220)
EPA + DPA + DHA	2,000 mg DHA, 560 mg EPA, 320 mg DPA	89 days, at least four times a week	Head trauma	≥18 years, American football player Treatment group, *n* = 31 Control group: *n* = 35	w-6/w-3 ratio ↓ EPA:AA ratio ↑ O3I ↑ Serum Nf-L levels ↓	(120)

w-3 PUFAs, omega-3 polyunsaturated fatty acids; EPA, eicosapentaenoic acid (20:5); DPA, docosapentaenoic acid (22:5); DHA, docosahexaenoic acid (22:6); mTBI, mild traumatic brain injury; WDI, weight drop injury; PPARγ, peroxisome proliferator-activated receptor gamma, NF-κB, nuclear factor kappa B; IL-1β, interleukin-1 beta; IL-6, interleukin-6; TNF-α, tumor necrosis factor alpha; RIP1, receptor-interacting protein 1; RIP3, receptor-interacting protein 3; MLKL, mixed lineage kinase domain-like protein; NSS, neurological severity score; TLR4, toll-like receptor 4; Nf-L, neurofilament light; GFAP, glial fibrillary acidic protein; HMBG1, high mobile group box 1; SIRT, sirtuin; RHT, repeated head trauma; MDA, malondialdehyde; MMP-3, matrix metalloproteinase-3; SCX, scleraxis; MMP-9, matrix metalloproteinase-9; AA, arachidonic acid; O3I, omega-3 index.

**TABLE 5:** Some studies on the mechanisms of action of vitamins and minerals in sports-related injuries.

Species/strain	Dosage	Duration	Sports injuries	Animal/human characteristics	Potential mechanism of action	References
Vitamin C	30 mM or 150 μM, in vitro	24 h	Tendinopathy	Human-derived tendon cells; Normoxia group: H2O2 group: 2 mM H2O2 Group C: 2 mM H2O2 + 150 μM Group HC: 2 mM H2O2 + 30 mM	ROS production in both doses ↓ Protection of actin filaments and cytoskeletons in the high vitamin C group TFAM, ATP5A, and type I collagen expression↑	(135)
Vitamin D	50.000 IU D3 [Administered to athletes with serum 25(OH)D levels < 30 ng/mL]	8 weeks, once a week, oral capsule	Stress fracture	NCAA Division I track and field team athlete (*n* = 118) 18–22 years	Incidence of stress fractures among cross-country running participants compared to previous seasons ↓	(142)
Vitamin D	−50.000 IU D3 [serum 25(OH)D levels < 20 ng/mL] −30.000 IU D3 [serum 25(OH)D levels < 20–39 ng/mL]	8 weeks, once a week, oral capsule	Stress fracture	NCAA Division I athletes (*n* = 802)	Risk of stress fractures in athletes whose vitamin D levels did not increase compared to athletes whose levels reached ≥ 40 ng/mL: 12.0% ↑ Athletes involved in indoor sports are more likely to have vitamin D deficiency, which increases their risk of stress fractures.	(143)
Vitamin D	50.000 IU D3	8 weeks, once a week, oral capsule	Stress fracture	University athletes Prospective cohort (*n* = 245) Retrospective cohort (*n* = 1,974)	Seasonal changes in vitamin D levels ↓ (regardless of gender and sport type) Risk of stress fractures in athletes taking vitamin D supplements compared to those not taking supplements: %1.67 ↑ (*p* > 0.05)	(144)
Vitamin D and calcium	2,000 mg Ca + 1,000 IU D3	12 weeks, twice a day, oral	Stress fracture	Marine recruits (*n* = 197) Ca + D bars = 945 mg Ca and 518 IU vitamin D3 (summer) 1,123 mg Ca and 469 IU vitamin D3 (winter) Placebo bars = ≤ 20 mg of incidental calcium and < 1.4 IU vitamin D3	BAP ve TRAP ↓ OCN ↓	(147)

Ca, calcium; NCAA, National Collegiate Athletic Association; TFAM, transcription factor A, mitochondrial; ATP5A, ATP synthase; BAP, bone alkaline phosphatase; TRAP, tartrate-resistant acid phosphatase; OCN, osteocalcin.

## 3 Biotics and their potential roles in sports injuries

The gut microbiome affects how nutrients are metabolized and plays a role in the immune system of the host, making it crucial for comprehending the metabolic and inflammatory reactions that take place following sports injuries through the gut-bone and muscle pathways (50). Sports injuries trigger the body’s stress response through tissue damage, immobility, and surgical interventions, initiating a stepwise process that begins wound healing. The healing process consists of stages of inflammation, regeneration and reconstruction that overlap with each other. In injuries to both soft and hard tissue, the recovery process begins with inflammation. The duration and severity of each stage varies based on many factors, such as the injury type, the individual’s nutritional status, and metabolic profile (6). Microbial metabolites and signaling molecules produced by the gut microbiota can influence muscle and bone healing processes by exhibiting pro-inflammatory, anti-inflammatory, and immunostimulatory effects (51). From this perspective, probiotics, prebiotics, and postbiotics constitute the most important microbiota-mediated approaches in balancing inflammation and supporting the healing process following injury (52).

Although acute and low-grade inflammation following sports injury is important for initiating the repair process, chronic inflammation can negatively affect healing by triggering catabolic processes in muscle and bone tissue (51). In particular, the activation of nuclear factor kappa-light-chain-enhancer of activated B cells (NF-κB) and mitogen-activated protein kinase (MAPK) pathways of activated B cells are activated, leading to an increase in cytokines such as tumor necrosis factor alpha (TNF-α), interleukin-1β (IL-1β), and interleukin-6 (IL-6), leading to the suppression of the PI3K-Akt-mTOR signaling pathway, which is one of the most critical anabolic pathways, and a decrease in protein synthesis (53). Additionally, during this process, the ubiquitin–proteasome system is activated via inflammation. Two important molecules involved in this process are Muscle RING-finger protein-1 (MurF-1) and Muscle Atrophy F-box protein (Atrogin-1), known as muscle-specific E3 ubiquitin ligases. The expression of ligases is regulated by myostatin, which negatively impacts muscle hypertrophy; thus, their levels rise in conditions like cachexia. Conversely, follistatin has a positive effect on muscle hypertrophy (54). Myostatin-induced muscle atrophy blocks the anabolic pathway IGF-1-PI3K-Akt and activates the transcription factor Foxo1, thereby accelerating muscle protein breakdown through increased atrogin-1 expression (53). In this context, the impact of the use of probiotics, prebiotics and postbiotics on muscle metabolism and inflammation-mediated mechanisms is being investigated.

The disruption of intestinal barrier integrity following injury is an intermediate mechanism in the intensification of the inflammatory process. This condition is commonly referred to as “leaky gut.” Increased intestinal permeability causes endotoxemia, which triggers the inflammatory response by increasing the passage of lipopolysaccharides (LPS) from the epithelial barrier into the systemic circulation. This inflammation activates catabolic pathways such as NF-κB in skeletal muscle via Toll-like receptor 4. NF-κB has been shown to contribute to muscle loss by triggering inflammation and blocking muscle fiber regeneration through its involvement in muscle protein breakdown (ubiquitin-proteasome pathway) (53).

The gut microbiota’s effect on muscle metabolism is also mediated through increased production of short-chain fatty acids (SCFAs), such as acetate, propionate, and butyrate, as well as secondary bile acids and specific amino acids like branched-chain amino acids. Short-chain fatty acids such as acetate, butyrate, and propionate produced by the gut microbiota improve insulin secretion by increasing glucagon-like peptide-1 (GLP-1) and Peptide YY (PYY) secretion mediated by G-protein-coupled receptor 41 (GPR41/43), suppresses NF-kB activation to affect inflammation, and regulates energy metabolism in muscle tissue through AMPK and PPAR-alpha/gamma activation. Energy metabolism in muscle tissue is regulated through the activation of AMP-activated protein kinase (AMPK) and peroxisome proliferator-activated receptors (PPAR-alpha and PPAR-gamma), which are crucial for maintaining cellular energy balance and regulating lipid and glucose metabolism. Additionally, insulin, insulin-like growth factor I (IGF-1), and certain amino acids stimulate anabolic processes in muscle and bone cells by activating the phosphoinositide 3-kinase (PI3K)/protein kinase B (AKT)/mechanistic target of rapamycin (mTOR) pathway. This pathway is vital for protein synthesis and cell growth, promoting muscle hypertrophy and enhancing bone density (52).

The effect of the intestinal microbiota on osteometabolism occurs through the regulation of inflammation and immune response (55). Immobilization during the follow-up of sports injuries increases proinflammatory cytokines, accelerating bone resorption via the RANKL/OPG signaling pathway. It has been demonstrated that interventions based on gut microbiota composition can prevent bone defects by suppressing the RANKL/OPG signaling pathway and through anti-inflammatory activity (56). Activation of the renin-angiotensin system (RAS) contributes to the development of osteoporosis by increasing the release of the receptor activator of nuclear factor-κB ligand (RANKL) from osteoblasts, whereas inhibition of this system may accelerate bone healing and remodeling (57). In an animal model investigating the effect of *Lacticaseibacillus casei* fermented milk, low serum levels of Ang II and RANKL were observed, suggesting that the osteoprotective effect may be mediated through suppression of RAS activity (58).

**FIGURE 2:**
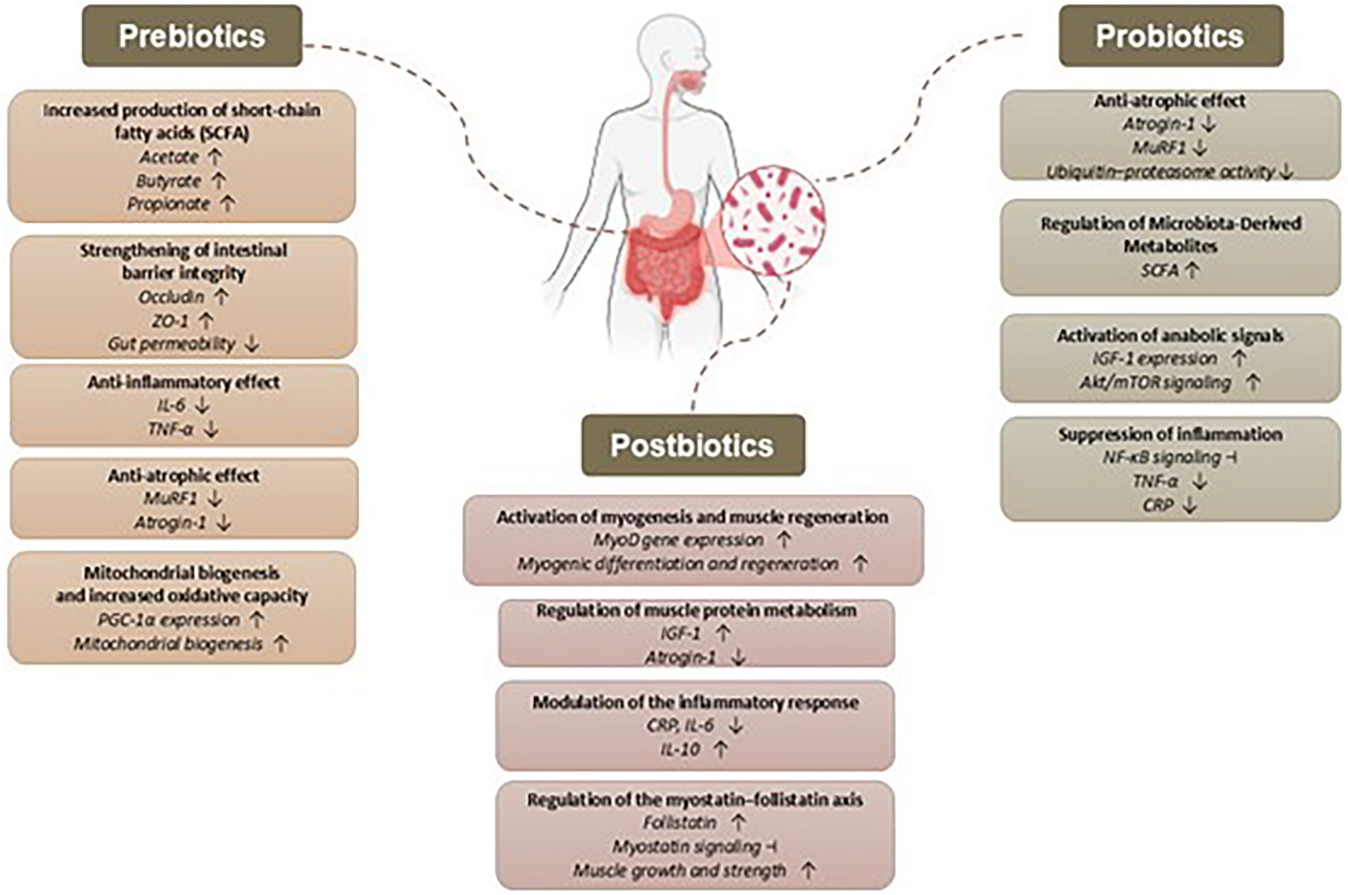
Potential mechanisms of action of biotics in sports-related injuries.

**FIGURE 3:**
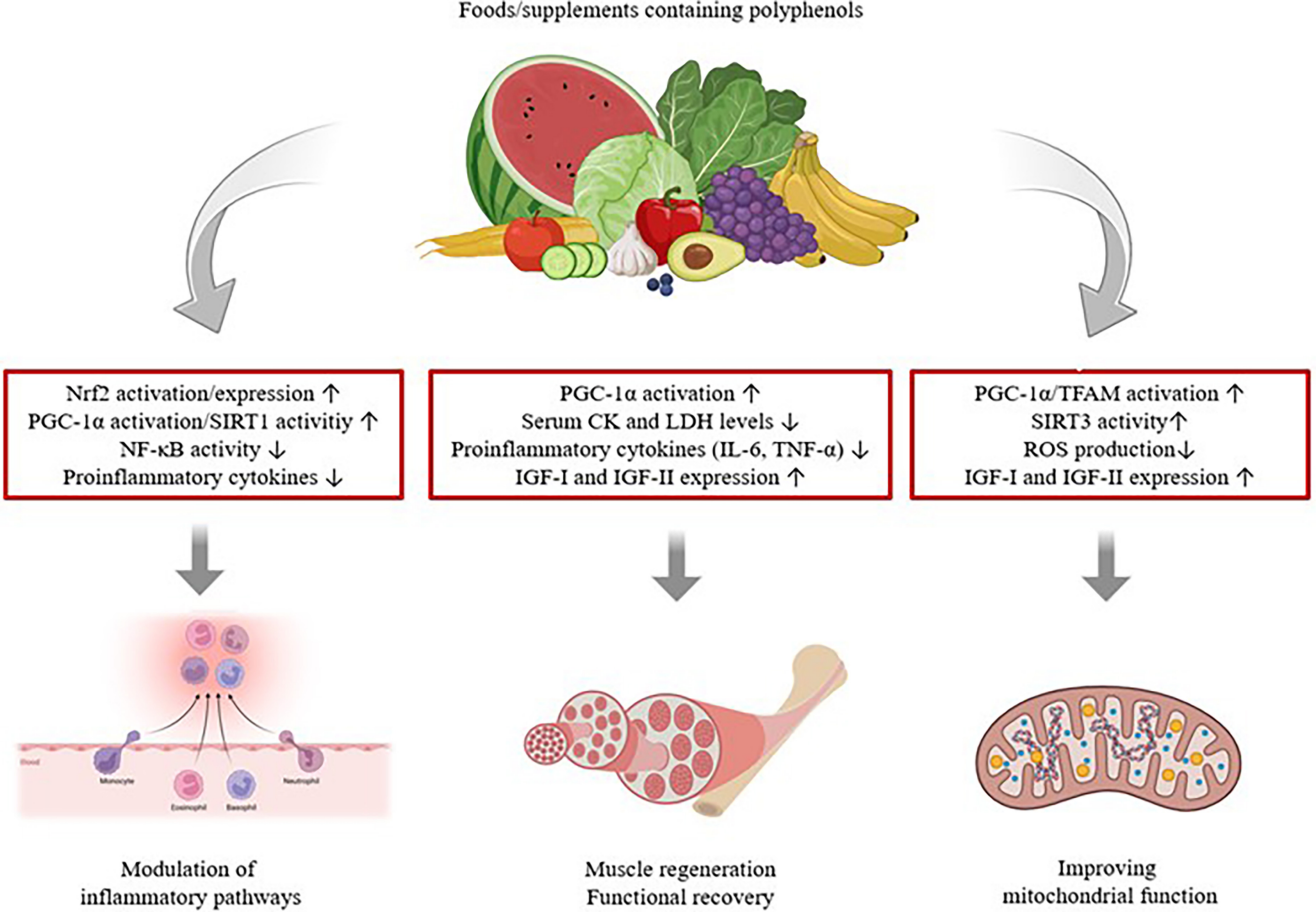
Effect of probiotic, prebiotic, and postbiotic interventions in sports-related injuries.

The microbiota regulates bone remodeling through hormones such as IGF-1 and immune cells. Short-chain fatty acids, which are beneficial metabolites produced by gut microbiota, play a crucial role in activating T cells, particularly regulatory T cells (Treg) and T helper 17 cells (Th17), within the intestinal environment. The proliferation of Treg cells in the bone marrow serves to dampen inflammatory responses, thereby promoting immune balance and effectively inhibiting the activity of osteoclasts, the cells responsible for bone resorption. This intricate interplay highlights the significant impact of microbial metabolites on immune regulation and bone health (59). *Clostridium butyricum* supplementation suppressed the proinflammatory IL-17A and Th17 response while enhancing the Treg response, thereby reducing inflammation in the bone marrow microenvironment (60). Considering the mechanisms mentioned above, the potential effects of biotics on healing have been examined under the categories of probiotics, prebiotics, and postbiotics. The potential benefits of probiotic, prebiotic, and postbiotic supplementation for injuries in sports may stem from the summarized biological mechanisms (Figures 2, 3).

### 3.1 Probiotics

The effect of probiotics on injuries has been studied in both human and animal studies, and these effects show that probiotics decrease inflammation, prevent the breakdown of muscle proteins, and support the proliferation of muscle cells (54, 55, 61, 62)

Preclinical studies have shown that probiotics may be effective in sports injuries by protecting joints, healing bone fractures, improving muscle damage, and reducing inflammation. In a rat model of anterior cruciate ligament severance, oral treatment with live and dead *Lactobacillus plantarum* GKD7 strains reported a decrease in IL-1β and TNF-α expression in synovial tissue and metalloproteinase (MMP)3 expression in cartilage, which is an enzyme that breaks down cartilage tissue (63). In rats with calcaneofibular ligament (CFL) injury, anscutaneous electrical nerve stimulation (TENS) modulated the gut microbiota, reduced IL-1β/NF-κB expression, and accelerated ligament injury healing (64). Optimal blood flow is essential for fracture repair. Increased systemic and local inflammation can hinder revascularization and impair fracture healing (65). Probiotics may reduce inflammation and promote revascularization of bone tissue. In mice with bone fractures, administration of *Akkermansia muciniphila* stimulated H-type vessel formation, promoting fracture healing more effectively than in a control group (66). In addition, studies conducted on animal models have reported that probiotic supplementation may limit immobilization-related muscle atrophy (67, 68). A study using *Lactiplantibacillus plantarum* strains found that probiotic supplementation suppressed atrogin-1 and MuRF-1 expression, thereby reducing inflammation-related muscle breakdown (69). Furthermore, a new probiotic combination consisting of *Lactobacillus acidophilus*, *Bacillus coagulans*, and *Streptococcus thermophilus* has been demonstrated to significantly lower myostatin levels and markers of muscle atrophy, including atrogin-1, MuRF1, and FoXO1 (70). When *A. muciniphila* EB-AMDK19 and *Faecalibacterium prausnitzii* EB-FPDK11 strains were administered to a mouse model of muscle atrophy for 4 weeks, there was a decrease in Atrogin-1, MuRF, and cathepsin L expression; decreased myostatin levels; increased expression of ZO-1 and anti-inflammatory IL-10 associated with intestinal barrier function; and decreased expression of pro-inflammatory IL-6 (67). These findings indicate that probiotics help protect muscle mass and performance through anti-inflammatory effects.

Research on the impact of probiotics on muscles function via metabolites show that: It has been reported that supplementation with *L. casei* Shirota strain, known to be effective on muscle function in animal models, causes changes in gut composition and increases short-chain fatty acid levels (acetate, butyrate, isobutyrate, etc.), thereby significantly reducing muscle mass and strength loss. (62). In another study conducted on animal models, supplementation with the *Lacticaseibacillus paracasei* EFEL6501 strain was reported to increase short-chain fatty acid levels (particularly acetate) and, in parallel, muscle width and muscle fiber section area (71). In their study, Chen et al. significantly improved bone and muscle loss induced by sodium dextran sulfate (DSS) exposure in mice through supplementation with *Bifidobacterium animalis* subsp. lactis A6 (B. lactis A6), which modulated the intestinal microbiota composition and increased butyrate-producing bacteria (55).

Human studies on sports injuries have mostly focused on exercise-induced muscle damage, and the limited studies that have been conducted have reported that probiotics may accelerate the healing process, slow down muscle strength loss after muscle damage, and speed up recovery. When reviewing human studies conducted, one study evaluated the use of a probiotic supplement containing *L. paracasei* PS23 in combination with omega-3 and leucine, while another study assessed the effect of *L. paracasei* PS23 alone on muscle mass and strength. Both studies reported the anti-inflammatory effect of probiotic supplementation and its favorable effect on muscle mass and function (72, 73). In a 17-week, double-blind, randomized controlled trial involving 19 elite male rugby players, the athletes were given daily probiotics (Ultra-biotic 60TM) and either *Saccharomyces boulardii* or a placebo. Based on their reports, muscle stiffness and leg fatigue were significantly reduced in the group receiving probiotic supplementation (74). In a randomized, double-blind study of 27 male marathon runners, probiotic supplementation (1 × 10^10^ CFU *L. acidophilus*, 1 × 10^10^ CFU *Bifidobacterium lactis* 30 days prior to the marathon) significantly reduced post-marathon lipopolysaccharide (LPS) levels (75). In a randomized, crossover study of 15 healthy men performing resistance training, daily supplementation with probiotics *S. thermophilus* FP4 and *Bifidobacterium breve* BR03, each encapsulated at a concentration of 5 billion live cells (AFU), starting 3 weeks prior to exercise causing muscle damage and continuing for 21 days, was reported to alleviate the reduction in range of motion following muscle damage (76).

The effects of probiotics are strain- and dosage-specific. Articles based on human studies included in this research mostly used doses ranging from 1 × 10^10^ to 6 × 10^10^ CFU/day, with intervention durations varying from 3 to 17 weeks. There is still no consensus on a definitive dosage and formulation for athlete recovery and injuries.

### 3.2 Prebiotics

Prebiotics are compounds that are selectively used by beneficial microorganisms in the gut microbiota, providing various health benefits (77). Prebiotics enhance the formation of short-chain fatty acids by being metabolized by useful gut bacteria like Bifidobacteria and Lactobacilli found in the colon. This process may help reduce system-wide inflammation and may also help regulate the immunity response (78). In sports injuries, systemic inflammation resulting from increased barrier permeability due to changes in gut microbiome composition delays healing. Prebiotics contribute to gut barrier healing by modulating microbial communities and metabolites (79).

Preclinical studies have shown prebiotics to be effective in suppressing muscle breakdown and in supportive roles following traumatic brain injuries. Traumatic brain injury, a significant risk factor for athletes, particularly in contact sports, alters the natural bacterial flora in the gut, initiating a chain reaction of gut cytokines that cause systemic inflammation (80). Prebiotic fiber supplementation, such as inulin, has been shown to prevent disruption of the gut flora and help maintain white matter integrity and cerebral blood flow in the brain (81). Supplements containing prebiotic fiber have been shown to prevent memory impairment after mild traumatic brain injury and to support the preservation of synaptic structure in the prefrontal cortex (82). In addition, prebiotics positively influence the gut microbial flora, enhancing muscle function and regeneration through their metabolites. One study found that rice bran supplementation increased ZO-1 and occludin levels, demonstrating enhanced mucosal barrier integrity, while decreased IL-6, TNF-α, and Hsp60 expression indicated suppressed inflammation (83). Similarly, another study demonstrated that inulin-enriched pea protein supplementation suppressed muscle protein degradation by the ubiquitin-protestasome path and enhanced muscle endurance through increased PGC-1α expression (78).

When human studies are examined, although studies in the literature are limited, the effects of prebiotics on reducing inflammation and healing muscle damage have been demonstrated. Supplementation with yeast-derived beta-glucan (250 mg), a prebiotic carbohydrate, resulted in a decrease in myoglobin levels, a marker of muscle damage due to intense exercise, and a decrease in inflammatory cytokine levels (84). Prebiotics are thought to be effective in the recovery from chronic sports injuries such as osteoarthritis. Supplementation with inulin, a prebiotic fiber, for 6 weeks (20 g/day) in individuals with osteoarthritis was found to reduce pain levels and increase hand grip strength (85).

Prebiotics have the ability to support the healing process of sports injuries. However, the type of prebiotic used, the dosage, and the duration of intervention in the studies are detailed in the table below, and there is considerable variability between studies. This difference limits comparisons between studies and the determination of the optimal dosage. In particular, the current evidence specific to sports injuries is quite limited, and therefore, future controlled studies are extremely important.

### 3.3 Postbiotics

Postbiotics can be described as “preparations consisting of inactivated microorganisms or parts of them that provide health improvements in the host.” As they do not contain live microorganisms, postbiotics do not pose an infection risk and can be obtained in a safe way from a wide variety of microorganisms (86). While probiotics directly provide live microorganisms to the gut microbiota, postbiotics are effective through inactivated microorganisms or metabolites produced by them. Unlike postbiotics, prebiotics are not products that directly exhibit biological effects, but rather nutritional sources that enable these effects to occur. Postbiotics contain metabolites produced by probiotics, such as exopolysaccharides, peptidoglycan, lipoteichoic acid, SCFA, and amino acids. It has been shown that non-viable bacteria and bacterial fractions can pass through mucus and stimulate epithelial cells more efficiently than live bacteria (87, 88). It is also suggested that postbiotics are associated with greater safety, standardization, and ease of preparation compared to live probiotics (89).

Preclinical studies have shown that supplementation with the postbiotic strain *L. plantarum* beLP1, isolated via kimchi, helps prevent muscle protein breakdown by regulating MuRF1 and Atrogin-1 expression (90). In another study, supplementation with heat-killed *L. plantarum* strains resulted in the suppression of p38 mitogen-activated protein kinase (p38) and extracellular signal-regulated kinase (ERK) phosphorylation in the MAPK signaling cascade, revealing an additional critical mechanism in the role of postbiotics in regulating muscle breakdown (86). Current data indicate that postbiotics have the ability to promote muscle function by reducing inflammation and inhibiting the breakdown of muscle proteins.

When human studies in the literature are examined; in a double-blind, 12-week placebo-controlled study in individuals aged 60 years and older, supplementation with pasteurized *A. muciniphila* HB05 (HB05P) significantly increased follistatin concentrations, compared to the control group, thereby inhibiting the effects of myostatin (54). In another study, the addition of heat-treated *L. paracasei* PS23 was found to reduce inflammatory cytokines like CRP and IL-6 and increase IL-10 levels. This demonstrates the effect of these postbiotics in supporting muscle tissue regeneration by reducing inflammation (73). In a double-blind, placebo-controlled clinical trial, 6 weeks of supplementation with *L. plantarum* TWK10 in healthy adult males resulted in significantly increased hand grip strength and muscle mass compared to a control group, while lower serum lactate and ammonia levels were reported during exercise and recovery periods (91). In another double-blind study, administration of a live *Lactobacillus paracasei* group (L-PS23, 2 × 10^10^ CFU/day) or a heat-killed *Lactobacillus paracasei* group (HK-PS23, 2 × 10^10^ cells/day) for 6 weeks resulted in a reduction in muscle strength loss and was shown to cause a decrease in blood creatine kinase and myoglobin levels (92). Furthermore, heat-killed *Lactobacillus paracasei* was found to be superior to the live form in terms of these effects. However, the fact that live bacteria can lose their viability depending on usage and storage conditions constitutes a significant limitation. The higher stability, safety, and shelf life of postbiotics may have provided an advantage. In the limited human studies conducted with postbiotic interventions, doses mostly ranged from 1 × 10^10^ to 3 × 10^10^ CFU/day, with intervention durations varying between 6 and 12 weeks. Clinical studies on determining the optimal dose range are quite limited.

Comprehensive clinical studies are required to establish the specific dosage, application duration, and efficacy of specific postbiotic strains for sports injuries. The key findings of studies examining the effect of probiotic, prebiotic, and postbiotic dietary intake and supplementation on the healing process are presented in Table 2.

## 4 Bioactive compounds and their potential roles in sports injuries

### 4.1 Polyphenols

Polyphenols represent a broad class of natural antioxidants widely distributed in dietary fruits and vegetables, as well as in microbial sources such as bacteria, fungi, and algae (93). In addition to their primary known antioxidant activity against peroxidation, polyphenols like resveratrol and flavonoids like quercetin and catechins have increasingly attracted attention in the field of sports medicine due to their ability to protect muscles from excessive exercise and oxidative stress (35, 94, 95).

**FIGURE 4:**
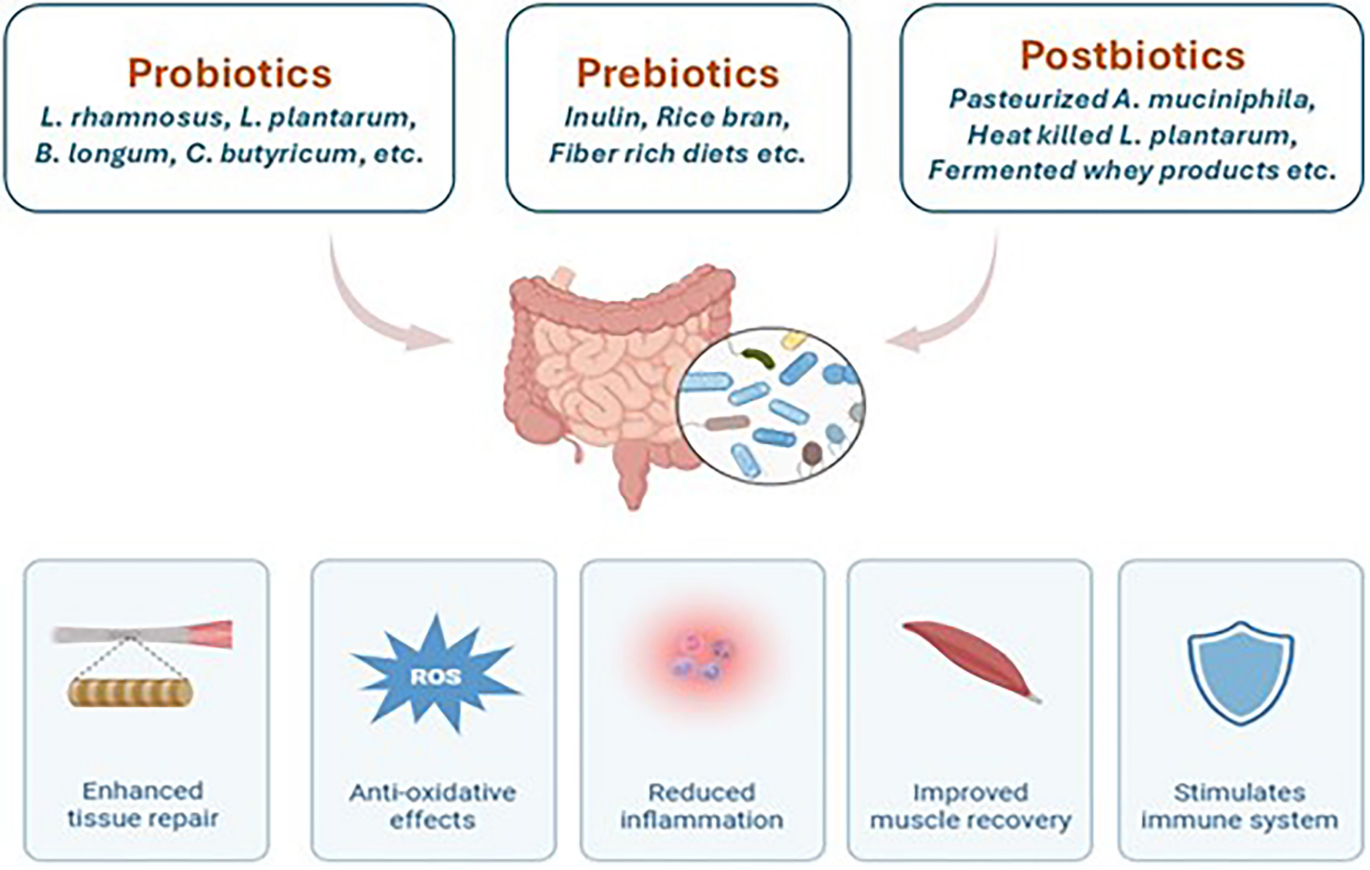
Potential mechanisms of action of polyphenols on sports injuries.

Recent studies have primarily focused on muscle damage, pain, and inflammation after matches or exercise (49, 96–98); there are very limited studies specifically on sports injuries (Table 3). Polyphenol supplements have been reported to have positive potential for influencing sports injuries by affecting metabolic processes related to redox homeostasis and skeletal muscle function (35, 96, 99). Moreover, it is stated that it promotes the enhancement of mitochondrial function, muscle regeneration, and the regulation of inflammatory pathways., as indicated in Figure 4 (35). A systematic review of the relationship between polyphenol supplementation and muscle recovery reported that supplementation taken for more than 7 days (60 mL per day, divided into two separate doses) may beneficial impact on team athletes’ muscle soreness and function (49). On the contrary, it is stated that a diet high in polyphenols could be a better approach than supplementation in preventing and treating injuries (100).

Polyphenols such as anthocyanins, quercetin, curcumin, cocoa flavanols, and resveratrol are associated with athletic performance (101–103). Anthocyanins reduce inflammation through their antioxidant effects and protect muscles against exercise-induced injury (101). Quercetin, a flavonol, is known to exhibit various bioactive effects with its strong antioxidant and anti-inflammatory properties. Although sufficient clinical evidence is lacking, quercetin has been suggested to possess the capacity to modulate exercise-induced oxidative stress and regulate inflammatory responses by affecting the Nrf2, PGC-1α/SIRT1, and NF-κB pathways (103). Nrf2 is a major transcriptional regulator that controls a variety of antioxidant enzymes involved in oxidative stress detoxification (104). PGC-1α is a key controller of mitochondrial biogenesis and the reactive oxygen species (ROS) stress response and is controlled by SIRT1/3, TFAM, and AMPK. PGC-1α and the immune response-regulating stress factor NF-κB are mutually regulated, such that reduced PGC-1α expression together with elevated oxidative stress leads to enhanced NF-κB activation (105). Additionally, quercetin supplementation has been reported to reduce serum levels of creatine kinase (CK), lactate dehydrogenase (LDH), and interleukin-6 (IL-6) in muscle injury and to increase the expression of insulin-like growth factor I (IGF-I) and insulin-like growth factor II (IGF-II), which have an important part in muscle regeneration (106).

Curcumin is one of the major phenolic compounds with the potential to enhance sports performance and recovery. Supplementing with curcumin has been shown to reduce inflammation and muscle damage brought on by exercise (107). Sajedi et al. (108), reported that two weeks of curcumin supplementation reduced indicators of muscle injury, including CK and LDH, after exercise (108). It has been suggested that curcumin may prevent muscle damage by inhibiting NF-kB activity, which plays a role in signaling associated with muscle injury (109). Notably, encapsulated or formulated curcumin has been demonstrated to lessen muscular inflammation and injury brought on by exercise (107, 110).

Like many polyphenols, resveratrol exhibits antioxidant and anti-inflammatory effects. It also interacts with PGC-1, activating SIRT1 and supporting energy metabolism and mitochondrial biogenesis (97, 111). Studies show that resveratrol increases muscle strength, improves fatigue tolerance, and promotes recovery (97, 112). Hsu et al. (113) found that resveratrol supplementation in mice may improve the muscular damage caused by contusions in rats by decreasing serum levels of uric acid, creatinine, LDH, and CK, as well as reducing myofibril damage (113).

It appears that the existing limited studies are insufficient in clarifying the effect of polyphenols on sports injuries due to their design, the diverse injuries they address, dose differences, and the fact that they are either animal or human studies (49, 114).

### 4.2 Omega-3 fatty acids

Omega-3 polyunsaturated fatty acids (ω-3 PUFAs) deficiency is a common danger for athletes and treating it can significantly improve their health. For athletes, the particular ω-3 PUFAs—eicosapentaenoic acid (EPA; 20:5), docosapentaenoic acid (DPA; 22:5), and docosahexaenoic acid (DHA; 22:6)—offer several benefits (115, 116). Research demonstrates that ω-3 PUFAs may reduce exercise-induced inflammation, diminish muscle damage and oxidative stress, decrease the oxygen expenditure during exercise, enhance immunological function, and favorably influence cognitive performance and cardiovascular health (116–118). Additionally, the ergogenic potential of ω-3 PUFAs in relation to sports injuries is also under investigation.

#### 4.2.1 Clinical studies

Current research indicates that the impact of w-3 PUFAs supplementation has been more extensively studied in relation to sports-related traumatic brain injuries than other injury types. After sports-related concussions, neuronal damage occurs alongside an increase in pro-inflammatory cytokine levels. Therefore, evaluating inflammation-related biomarkers is crucial for differentiating and treating concussions (119, 120). A study conducted by Mullins et al. (121) found that supplementation with 2.5 g of DHA and 1.0 g of EPA in American football players increased the concentration of plasma phosphatidylcholine derivatives, which can cross the blood-brain barrier, after 4 weeks. This indicates that such supplements may confer advantages regarding sports-related concussions or cerebral injuries (121). Raikes et al. (122) reported no significant difference in serum Nf-L levels, considered a biomarker of axonal damage, between the placebo and the both EPA and DHA treatment group in their study with American football players. Additionally, no significant effect of DHA + EPA supplementation was observed in volumetric brain measurements. However, DHA + EPA mediated the preservation of the brain’s microstructural integrity (122). Oliver et al. (123) showed that DHA supplementation in American football players resulted in a dose-independent decrease in Nf-L levels in all three groups after head trauma (123). Heileson et al. (120) reported that w-3 (EPA + DPA + DHA) supplementation in American football players could prevent neuroaxonal damage by reducing Nf-L levels. Additionally, the serum w-6/w-3 ratio decreased while O3I increased, thereby reducing systemic inflammation (120). A systematic review reported that w-3 PUFAs may have beneficial impacts on adverse outcomes such as cognitive function, sleep disorders, and anxiety after mTBI (124).

Currently, there are few human studies examining the impacts of w-3 supplementation on sports injuries, the results are frequently inconsistent. This inconsistency may be due to various factors, including the research population’s features, the duration of the research, the specific form of w-3 fatty acid used, and the dosage administered. It’s crucial to remember that only about 5%–10% of alpha-linolenic acid (ALA) can be converted into EPA, while only 2%–5% can be transformed into DHA. Factors such as genetic diversity and gender differences in enzyme activity can influence this conversion process, thus impacting an individual’s overall w-3 PUFAs production status (116). Triglycerides, free fatty acids, ethyl esters, re-esterified triglycerides, and phospholipids are among the different types of w-3 PUFAs that can be found in W-3 supplements. The body has varying levels of W-3 fatty acid bioavailability. Compared to ethyl esters, esterified triglycerides, natural triglycerides, and free fatty acids often have slightly higher bioavailability. Furthermore, compared to triglycerides, phospholipids have greater bioavailability. As a result, while eating all types of W-3 raises EPA and DHA levels, their efficacy varies according to their bioavailability (116, 125).

Evaluating athletes’ w-3 status at both baseline and the end of training is essential, along with assessing indices such as the omega-3 index and the AA/EPA ratio. This evaluation is important for understanding various types of injuries (118, 126). Therefore, it may be beneficial to examine the effects of w-3 supplementation in relation to these indices across different sports-related injuries. Additionally, the development of dose-dependent gastrointestinal symptoms should be monitored (123). In conclusion, there are currently no clear recommendations regarding effective and safe dosages or durations for omega-3 supplementation (127). Thus, the findings indicate that more research is necessary.

#### 4.2.2 Animal studies targeting mechanisms

**FIGURE 5:**
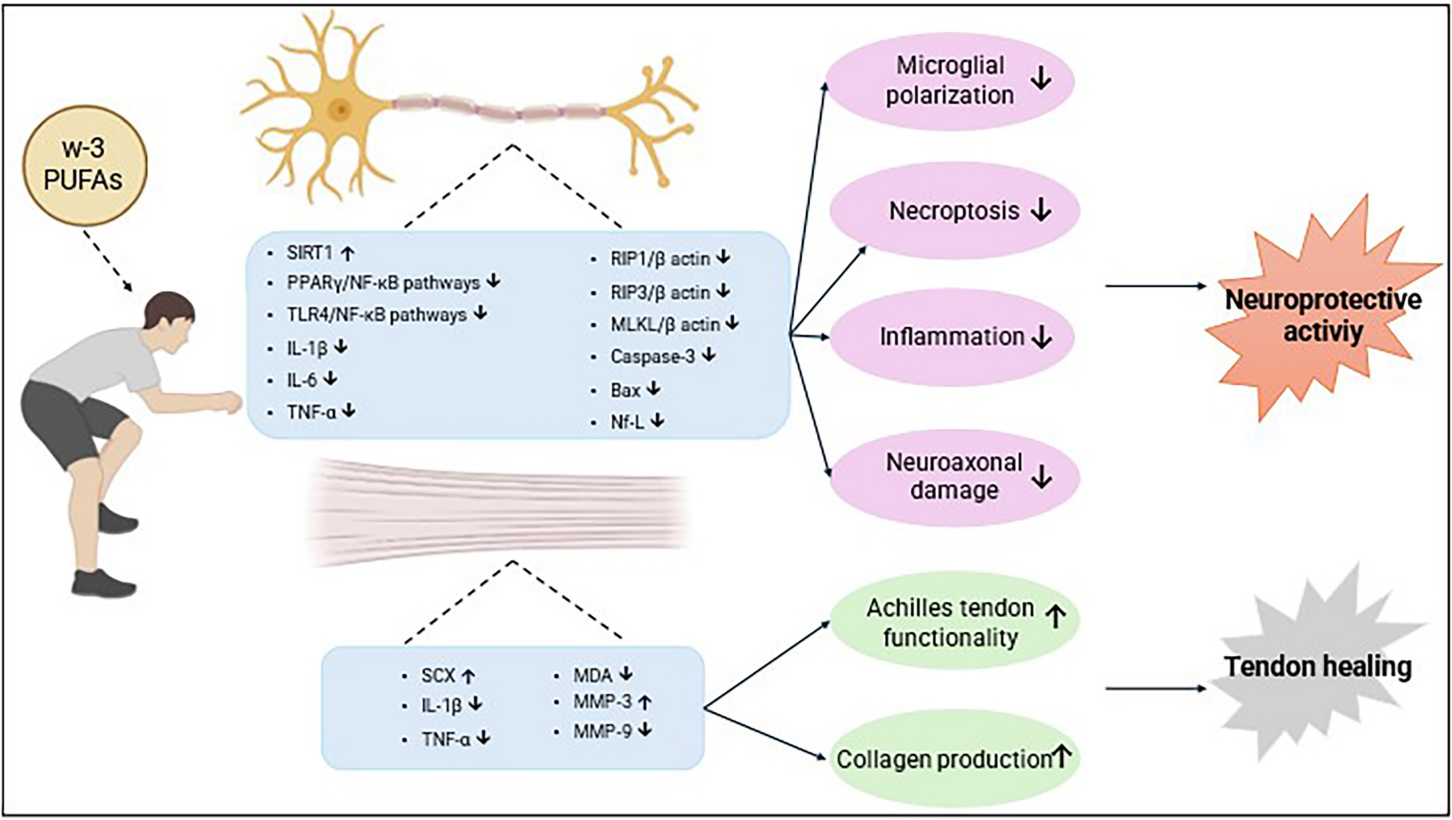
Potential mechanisms of action of w-3 PUFAs in sports-related injuries.

The potential positive effects of w-3 supplementation for injuries in athletes may arise from a combination of biological mechanisms (Figure 5). Wu et al. (128) reported that administering w-3 fatty acids after mTBI reduced neuroinflammation and suppressed necroptosis in rats. The anti-inflammatory proporties were evident through a decrease in levels of NF-κB, IL-1β, IL-6, and TNF-α in the hippocampus, achieved via the PPARγ/NF-κB signaling pathway. The levels of RIP1/β actin, RIP3/β actin, and MLKL/β actin, which rise in the brain cortex after mTBI, were significantly reduced, indicating the anti-necrosis effects. Additionally, omega-3 supplementation reduced cerebral edema by lowering brain water content 72 h after mTBI, resulting in a significant decrease in the Neurological Severity Score (NSS). In conclusion, the neuroprotective impacts of w-3 PUFAs following mTBI are due to their modulation of anti-necrotic and anti-inflammatory pathways (128). Neuroinflammation can also result from long-term microglia activation. TBI-induced microglial activation and the ensuing inflammatory response are significantly influenced by HMGB1 release. Reducing microglial polarization may be a protective factor in reducing chronic inflammation after TBI. w-3 PUFA supplementation has been demonstrated to exert a neuroprotective effect by regulating microglial polarization via SIRT1-mediated deacetylation of the HMGB1/NF-κB pathway, thereby inhibiting the inflammatory response following TBI (129). A similar study concluded that w-3 supplementation inhibits HMGB1 expression in microglia in lesion areas after TBI and may block the inflammatory response following TBI by inhibiting HMGB1-mediated TLR4/NF-κB pathway activation (130). Lust et al. (131) reported that w-3 PUFAs (3%) administered to male rats prior to mTBI helped prevent structural brain damage. Additionally, w-3 PUFAs improved neurological function within 7 days after mTBI (131). Additionally, following WDI injuries, w-3 PUFA demonstrated a neuroprotective impact on the recovery of cognitive, motor, and behavioral skills; this beneficial effect was mediated by a decrease in brain cellular damage (132). Irani et al. (133) reported that aerobic exercise combined with w-3 PUFA reduced inflammation in rats with tendon damage and supported Achilles tendon regeneration by increasing collagen production and cell differentiation (133).

In conclusion, w-3 fatty acids may help decrease inflammation and suppress tissue death, providing a neuroprotective effect following traumatic brain injury. Additionally, they may speed up the healing process by promoting tendon regeneration (Table 4). However, these findings are based on studies conducted with rats, so the same effects might not occur in athletes. Therefore, further research involving athletes is necessary, and the specific mechanisms by which w-3 fatty acids operate need to be elucidated.

### 4.3 Vitamins and minerals

#### 4.3.1 Vitamin C

Because it functions as a cofactor for the enzymes prolyl hydroxylase and lysyl hydroxylase, vitamin C is essential for the repair of connective tissues (134). Additionally, it acts as a potent antioxidant, assisting in the prevention of advanced glycation end products (AGEs) and oxidative stress (135). Conversely, a lack of vitamin C has been associated with decreased procollagen production and decreased hydroxylation of proline and lysine residues, which may impede the repair of tendons (136). *In vitro* studies have shown that exposing human tendon cells to normal (150 μM) and high (30 mM) doses of ascorbic acid leads to decreased cell death and reduced oxidative stress. However, only high dosages of vitamin C efficiently prevented disruptions in the cell cytoskeleton (135). The impact of vitamin C on wound healing after bone, tendon, and connective tissue injuries was assessed in a comprehensive study. According to recent research, vitamin C increases the production of type I collagen, may assist lower oxidative stress parameters, and accelerates bone repair after fractures in preclinical experiments (134). Considering that 60%–85% of the primary molecular component of tendons is type I collagen, this could be significant for the regeneration of tendon cells after injuries in athletes (136).

Following a TBI, ascorbic acid (vitamin C) supplementation may be an effective strategy for preventing or reducing secondary brain injury. This can be achieved by inhibiting lipid peroxidation in the brain, decreasing ROS, suppressing pro-inflammatory mediators, stabilizing the endothelium, and minimizing cerebral edema (137). Additionally, vitamin C has been shown to upregulate the expression of the sodium-dependent vitamin C transporter 2 (SVCT2) and cysteine-rich protein 3 (CSRP3) in muscle cells in rats. The increase in CSRP3 levels has been linked to the differentiation of C2C12 cells, a process that facilitates interaction with myogenic regulatory factors, including MyoD and MyoG, which are involved in muscle repair and regeneration (138). Vitamin C may positively affect sports-related injuries due to its collagen-boosting properties, as well as its antioxidant and anti-inflammatory effects. However, the current evidence is limited and insufficient to support the use of vitamin C supplementation specifically for these injuries. Furthermore, most studies conducted so far have not focused on athletes. Therefore, more research is needed to better understand the role of vitamin C supplementation in sports injuries, particularly in relation to athlete physiology.

#### 4.3.2 Vitamin D and calcium

Vitamin D is an important vitamin that garners attention in various aspects of sports nutrition. It has multiple functions, including as controlling the metabolism of calcium and phosphorus, supporting the immune system, synthesizing proteins, and controlling the cell cycle. Furthermore, vitamin D contains anti-inflammatory qualities that may elhp prevent injuries associated to sports (139). A study by Rebolledo et al. (140) found that approximately 59% of elite soccer players suffered from vitamin D deficiency (140). Research indicates that a deficiency in vitamin D can negatively impact athletic performance and raise the risk of injuries, particularly by affecting endurance (139, 141). A meta-analysis found no significant correlation with other musculoskeletal injuries, however 25-hydroxyvitamin D levels below 30 ng/mL were associated with an increased risk of stress fractures (139). Furthermore, another study showed that vitamin D deficiency in athletes increased the likelihood of lower extremity strains or abdominal muscle injuries by 1.86 times and the risk of hamstring injuries by 3.61 times (140). Maintaining sufficient vitamin D levels is therefore essential for avoiding sports-related injuries.

According to a recent meta-analysis, vitamin D supplementation may improve anaerobic power, strength, and aerobic endurance in elite athletes. However, contradictory data regarding bone health and injury risk indicate that more study is required (24). Williams et al. (142) administered oral vitamin D3 supplementation at a dosage of 50,000 IU once weekly for 8 weeks to American football players with serum 25(OH)D concentrations below 30 ng/mL. The cross-country team’s stress fracture rate significantly decreased as a result of this intervention, going from 7.51% to 1.65% with vitamin D treatment (142). Millward et al. (143) also demonstrated that vitamin D supplementation in athletes with vitamin D concentrations below 40 ng/mL decreased the stress fracture rate by 12.0% (143). Conversely, another study indicated that calcitriol, administered to all athletes at the same dose and duration, did not significantly affect the stress fracture rate (144). Additionally, the reasons of vitamin D deficiency in athletes require thorough evaluation. For instance, Black professional athletes who compete indoors are at a greater danger of vitamin D deficiency and stress fractures (143, 145). Seasonal changes, particularly during winter, also influence vitamin D deficiency and, consequently, the risk of injury (142).

Bone mineralization depends on calcium, and the body’s calcium levels are controlled by calcitriol, calcitonin, FGF-23, and plasma calcium concentration (146). Consequently, calcium and vitamin D are closely interconnected. In a study focused on stress fractures, participants were given bars containing 2,000 mg of calcium and 1000 IU of vitamin D twice a day for 12 weeks during military training. Researchers evaluated various parameters related to bone health. The supplemented group showed lower levels of osteocalcin (OCN), tartrate-resistant acid phosphatase (TRAP), and bone alkaline phosphatase (BAP). Additionally, an rise in 25(OH)D vitamin levels was noted in this group. However, the impact of supplementation on bone tissue appeared to be limited and varied according to the season. Notably, the observed declines in BAP and TRAP may indicate a decrease in bone remodeling, which could improve the equilibrium between mineralization and bone resorption and strengthen bones (147).

The transcription of target genes necessary for calcium and phosphorus metabolism, bone mineralization, and remodeling is crucially regulated by the vitamin D receptor (VDR). These genes include cytochrome P450, FGF-23, calcitonin gene-related peptide (CGRP), and receptor activator of nuclear factor kappa-β ligand (RANKL). Additionally, the VDR interacts with important signaling pathways for bone formation, such as the Wnt/β-catenin signaling pathway, PPARγ, and the estrogen receptor (ER) (148). As a result, the interaction between vitamin D and VDR promotes the differentiation and formation of osteoblasts while inhibiting the production of osteoclasts. Additionally, vitamin D improves calcium and phosphorus absorption, which is associated with higher bone mineral density (149, 150). These mechanisms may help reduce the risk of stress fractures by supporting bone formation. It has also been reported that VDR targets genes related to muscle structure and energy metabolism, such as ACTA1 and SLC25A4, following anterior cruciate ligament reconstruction (151). Together with muscle troponin I and T, vitamin D is essential for increasing the production of myogenic factors such MYOD, MYOG, MYH1, and MYC type II. Additionally, it promotes muscle cell differentiation, development, and regeneration by upregulating the production of IGF, FGF, and TGF-β (152). These findings suggest that preventing vitamin D deficiency and supplementing with calcium, whether individually or in combination, may help prevent sports injuries related to muscle and bone tissue or aid in recovery after an injury (Table 5). However, further prospective studies are needed in this area.

#### 4.3.3 Magnesium

There is no specific study on magnesium and sports injuries. However, magnesium is a cofactor of ATP and therefore plays a role in many enzymatic reactions related to energy production, storage, DNA, and RNA. It reduces oxidative stress and inflammation (153). According to reports, magnesium aids in myogenesis, enhances blood, muscle, and brain glucose utilization, and inhibits the production and buildup of lactate in muscles (154, 155). Additionally, magnesium increases osteoblastic activity by enhancing calcium retention in bone (156). Due to these mechanisms, magnesium supplementation has been reported to be an effective method for repairing muscle damage and stress fractures (157). However, it is important to accurately measure each person’s magnesium levels. A meta-analysis found that while magnesium supplementation had no noticeable effect on muscular performance in athletes and physically active people with comparatively higher magnesium levels, it did improve muscle strength and power in older adults and alcoholics with magnesium deficiencies (158). Magnesium may therefore indirectly lower the risk of injury and enhance the healing process in athletes who are deficient in the mineral. Preclinical and clinical studies are yet to be conducted in this field.

### 4.4 Amino acids and peptides

The impact of amino acids and amino acid-derived peptide interventions on athletes during immobilization periods for rehabilitation after acute injuries remains uncertain (159). Nonetheless, supplementation with these compounds could potentially enhance tissue recovery at the injury site and aid the healing process (160) (Table 6).

**TABLE 6:** Some studies on the mechanisms of action of amino acids and peptides in sports-related injuries from both animals and clinical trials.

Species/Strain	Dosage	Duration	Sports injuries	Animal/human characteristics	Potential mechanism of action	References
ALCAR	Subcutaneous injection, 600 mg/kg/day	For prophylactic purposes, 14 days before the first head injury, 15 blows, 24 days	RHT	Male C57BL/6J mice; RHT group with no ALCAR treatment, RHT with ALCAR	RHT + ALCAR group, mRNA levels of TARDBP and CCL11 in the prefrontal cortex ↓ (to baseline) Hippocampal MAPT, TARDBP, and GFAP mRNA levels ↓ (return to baseline)	(166)
L-carnitine	Intraperitoneal, 300 mg/kg	7 days	Achilles tendon injury	Group 1: normal (Achilles tendon injury and the applications listed below were performed in all groups except Group 1) Group 2: +Surgical repair only Group 3: +Physiological serum Group 4: +L-carnitine	Matrix metalloproteinase level ↔ Maximum strength and durability ↑	(167)
L-carnitine	Intraperitoneal, 100 mg/kg	1, 6, 24, and 48 h after the procedure	TBI	Male C57BL/6 mice Group 1: control group (*n* = 10) Group 2: craniotomy group (*n* = 9) Group 3: TBI + saline (*n* = 10) Group 4: TBI + mildronate (*n* = 9) Group 5: TBI + L-carnitine (*n* = 10)	Mitochondrial biogenesis ↑ IL-1β, IL-6, TNF-α, PTGS2, GFAP ↑ *Tenericutes* abundance ↑	(165)
Creatinine	Loading phase = 20 g/day = 4*5 g Maintenance phase = 5 g/day, 1*5 g	42 days (first 5 days loading phase + Next 37 days maintenance phase)	Flexor hallucis longus tendinopatisi	12–18 years Paletted adolescent swimmers Creatinine (CR) group (*n* = 9, M = 5, F = 4) Placebo (PL) group (*n* = 9, M = 5, F = 4)	During the 2-week immobilization period, there was a lower rate of decrease in segmental lean mass (CR: 5.6%, PL: 8.9%, *p* < 0.001) During the rehabilitation period (4 weeks), a higher rate of increase in segmental lean mass (CR: 5.5%; PL: 3.8%, *p* < 0.001) Pain severity ↓ PFT ↑ CK ↓	(173)
Collagen peptides	Collagen hydrolysate-based product; 4,500 mg before training, 2,500 mg after training	3 months	Tendon structure	Elite figure skaters (*n* = 18, M: 9, F: 9)	Tendon thickness in the patellar and peroneal tendons ↑ Proximal and medial APD of the patella and peroneal tendons ↑ Tendon cross-sectional area ↔	(183)
Collagen peptides	2.5 g/day, once daily	The duration is 6 months, with group changes occurring after 3 months	Achilles tendinopathy	Intervention group (*n* = 9, M = 5, F = 4) Placebo group (*n* = 9, M = 7, F = 2) Calf strengthening exercises were implemented for both groups.	Achilles tendon pain and functional recovery Number of people returning to running activities ↑	(185)
Mucopolysaccharide, vitamin C, collagen peptides	Gastric gavage; Mucopolysaccharide: 220 mg Vitamin C: 30 mg) Hydrolyzed type I collagen: 40 mg; 7.2 mg/kg	3 weeks, every day	Achilles tendon injury	Male Wistar albino rats; control group (*n* = 7) experimental group (*n* = 7)	PCNA, TGF-β1, and COL1 ↔	(186)

ALCAR, acetyl L-carnitine; RHT, repeated head trauma; MAPT, microtubule-associated protein tau; TARDBP, TAR DNA-binding protein; CCL11, C-C motif chemokine ligand 11; PFT, plantar flexion peak torque; CK, creatine kinase; COL1, collagen type I; PCNA, proliferating cell nuclear antigen; TGF-β1, transforming growth factor β1; APD, anteroposterior distance; GFAP, glial fibrillary acidic protein; PTGS2, prostaglandin-endoperoxide synthase 2; IL-1β, interleukin-1 beta; IL-6, interleukin-6; TNF-α, tumor necrosis factor alpha.

#### 4.4.1 L-carnitin

L-carnitine is a product of amino acids that is synthesized endogenously in the liver, kidneys, and brain (∼25%) or obtained from animal-derived foods (∼75%) (161). Long-chain fatty acids are transported into the mitochondrial matrix for utilization in energy synthesis by L-carnitine, which also contributes to their β-oxidation (162). There is evidence that L-carnitine may have a beneficial impact on muscle injury and recuperation after exercise (163). However, there is limited evidence that can be linked to sports-related injuries. It has been reported that 1.5 mM of L-carnitine in drinking water administered to rats for 2 weeks immediately after traumatic brain injury reduced cortical contusion after 6 weeks and may improve neurological function and tissue integrity by reducing oxidative stress. However, in this study, L-carnitine was not administered alone but in combination with exendin-4 (164). Gureev et al. (165) reported that L-carnitine intervention (100 mg/kg, intraperitoneal) in rats with traumatic brain injury promoted mitochondrial biogenesis and thus had a protective effect against mitochondrial DNA damage (165). In another rat study, preventive administration of acetyl L-carnitine (ALCAR), a type of carnitine supplementation, decreased the chronic effects of repeated head trauma, improved spatial learning and memory, and provided neuroprotective effects (166). On the other hand, it has been reported that L-carnitine does not cause a significant difference in tendon repair but supports maximum strength and endurance after Achilles tendon injury (167).

Carnitine’s beneficial benefits could be attributed to its control of the PTEN/Akt/mTOR signaling pathway, which prevents oxidative stress, increases axonal plasticity, and improves oligodendrocyte myelination of axons (168). Furthermore, L-carnitine is an activator of the Nrf2/ARE signaling pathway, which may help reduce oxidative stress in the injured area of the brain and activate antioxidant defenses (169). But according to Gureev et al. (165), L-carnitine may raise proinflammatory cytokine levels such IL-1β, PTGS2, GFAP, IL-6, and TNF-α, which would cause inflammation. This impact may be primarily caused by carnitine’s capacity to boost the number of *Tenericutes* in the gut microbiota. This is due to the fact that TMA is converted to TMAO in the gut microbiota by bacteria belonging to the phylum *Tenericutes* (165). As a result, different physiological situations may have varied effects from L-carnitine. This circumstance emphasizes the necessity of expanding preclinical and clinical research on the therapeutic application of L-carnitine in sports injuries and elucidating the mechanism.

#### 4.4.2 Creatine

Increasing muscle mass is crucial for enhancing muscular strength and endurance and regaining lost performance in athletes, particularly in the advanced stages of rehabilitation after muscle injuries. The body may naturally produce creatine, often referred to as α-methylguanidine acetic acid, from the amino acids glycine, arginine, and methionine (170). It has been shown that giving athletes a creatine supplement (5–9 g per day, for as long as 32 weeks) a few hours before or right before training, or a few hours after or right after a training session, will boost muscle mass and assist in muscle regeneration (23). A meta-analysis study reported that combined exercise and creatine supplementation increased lean body mass in men regardless of age, but its effectiveness was lower in women. Additionally, it was reported that creatine had no significant effect on muscle repair without exercise (171). Backx et al. (172) demonstrated that creatine loading did not improve muscle fiber size or muscle mass in the absence of short-term muscle use and did not prevent muscle strength loss (172). However, there is limited evidence that creatine is effective for injuries directly related to sports. Juhasz et al. (173) tested the effectiveness of creatine in excessive tendon use injuries in a study conducted with adolescent swimmers. Consequently, creatine improved muscle mass and maximal torque of muscles during plantar flexion to a greater degree, enhanced muscle regeneration, and quickly reduced pain intensity when compared to the placebo group (173).

Creatine may aid in muscle repair after sports injuries in several ways. Based on the CC genotype and the C allele of the AMPD1 gene, which are linked to muscle performance, a study involving professional soccer players discovered that creatine supplementation, which consisted of a loading dose of 20 g of creatine monohydrate daily for 5 days, followed by a maintenance dose of 3–5 g daily for 7 weeks, affected muscle mass. Additionally, it was found that athletes with a low total genotype score were 9.4 times more likely to sustain a muscle injury (95% CI: 4.535–19.425; *p* < 0.001) (174). By increasing cellular hydration, creatine’s osmotic qualities may function as an anabolic stimulant for protein synthesis. Certain protein kinases and signaling pathways, like mTOR, are responsible for this. By modifying myogenic transcription factors and insulin-like growth factor-1 (IGF-1), creatine may also promote the differentiation and proliferation of muscle cells. Furthermore, by lowering oxidative stress and inflammation, which includes lowering TNF-α and IL-6 levels in muscle cells, it can prevent muscle catabolism and encourage muscle mass development (174–176). Moreover, creatine helps prevent muscle atrophy by reducing proteolysis associated with Atrogin-1 and MuRF-1 (177). It also compensates for the decrease in Glucose Transporter-4 (GLUT4) levels during immobilization and assists in increasing GLUT4 during rehabilitation (178).

#### 4.4.3 Collagen

The main element of the extracellular matrix, collagen is made up of glycine, proline, and hydroxyproline and is essential for controlling and regenerating connective tissue (179). Bioactive peptides and amino acids are produced during the hydrolysis of collagen peptide supplements. Collagen-derived prolyl-4-hydroxyproline, along with alanine and glycine, promotes cell division. Additionally, when combined with leucine, it enhances collagen secretion in preosteoblast cells (180). These bioactive peptides, in particular, have been documented to have positive impacts on tissue injuries (181). According to a comprehensive review, supplementing with 15 g of collagen peptide daily for 8 weeks may help avoid sports injuries by increasing tendon cross-sectional area (182). Giannini et al. (183) reported that the application of collagen hydrolysate, with doses of 4,500 mg before and 2,500 mg after training in elite skaters, increased both the thickness of the patellar and peroneal tendons and the anterior-posterior distance over a 3-month period. Nevertheless, the tendon cross-sectional area did not significantly change as a result of this treatment. This discovery may be used as a prophylactic measure to preserve tendon health, particularly in high-impact sports (183). Furthermore, a meta-analysis found that supplementing with 15 g of collagen daily improved joint function, increased collagen synthesis rates, and improved muscle growth and strength (184). Praet et al. (185) also found that combining collagen peptides with calf strengthening exercises reduced Achilles tendon pain and improved overall functionality. Although participants in the collagen peptide group did not fully return to pre-injury levels, a higher number were able to resume running sports compared to those who did not receive the supplementation (185). Proliferative cell nuclear antigen (PCNA), transforming growth factor β1 (TGF-β1), and type I collagen were among the markers in the repair area that Gemalmaz et al. (186) found were not significantly affected by supplementation with mucopolysaccharide (220 mg), vitamin C (30 mg), and hydrolyzed type I collagen (40 mg). This indicates that the supplementation did not promote cell proliferation or tissue regeneration (186). On the other hand, the positive effects observed in tenocytes may be attributed to various mechanisms. For instance, research on adult tendon cells revealed that the collagen-derived dipeptide prolyl-4-hydroxyproline (Pro-Hyp) supports cellular homeostasis through active β1-integrin and enhances actin-based cell movement generated by lamellipodia (180). Furthermore, hyaluronic acid and collagen were found to downregulate CD14 in cells derived from human Achilles tendon, which is linked to decreased inflammation in macrophages. This also positively influenced CD44 and type I collagen, aiding in the remodeling of the extracellular matrix. Additionally, increased cell membrane vesiculation and cell size further facilitated the proliferation of tenocyte cells (187).

Collagen peptides have been documented to have beneficial effects on bone healing and the prevention of muscle loss. Collagen peptides can improve indicators of bone metabolism and increase bone mineral density in the femoral neck and spine, according to a recent meta-analysis. These benefits may be enhanced when combined with vitamin D and calcium (188). In particular, Pro-Hyp controls Runx2 activity by directly binding to Foxg1 during osteoblast differentiation. This interaction prevents Foxg1 from affecting Runx2, thereby supporting osteogenic differentiation (189). Rats with muscle atrophy were given collagen (0.25 or 0.5 g/kg) for 4 weeks. This resulted in an increase in calf muscle volume and a restoration of the cross-sectional area of muscle fibers. Collagen’s capacity to suppress Smad2 phosphorylation and myostatin mRNA expression may be responsible for this advantageous effect. TGF-β activates Smad2, a transcription factor involved in intracellular signal transmission. Collagen also activated the AKT/mTOR signaling pathway, which is essential for the creation of muscle proteins (190). The phosphatidylinositol 3-kinase-protein kinase B (PI3K-Akt), mitogen-activated protein kinase (MAPK), and mechanistic target of rapamycin (mTOR) pathways were found to be activated in skeletal muscle when recreational athletes took 15 g of collagen during acute resistance exercise (191). It is known that these pathways have a role in the remodeling of muscle cells. While current findings indicate that collagen peptides may have beneficial effects for athletes recovering from muscle and bone injuries, most studies do not focus specifically on athletes and often explore indirect effects. As a result, the clinical evidence in this area remains limited, highlighting the need for further research that takes athlete physiology into account.

#### 4.4.4 Arginine and glutamine

L-arginine contributes to the urea cycle, aids in the synthesis of collagen, and is an essential substrate for the production of nitric oxide (NO). Collagen production, tissue and wound healing mechanisms, and cell proliferation are all regulated by nitric oxide (192, 193). Many cells, including lymphocytes and fibroblasts, use glutamine as an energy source to proliferate. It is known that under specific physiological circumstances, such as stress and trauma, arginine and glutamine become vital. As a result, getting glutamine and arginine from food is crucial (193). L-arginine is also intimately associated with bone metabolism. Arginine supplementation at a level of 10 mg/kg was observed to increase trabecular bone volume and bone mineral density in rat research. However, sex hormones had a big impact on this effect and affected the outcomes (194).

Oral L-arginine supplementation decreased defect areas, increased the number of osteoblasts and osteoclasts, and subsequently accelerated the rate of bone production in patients with fractures, according to a systematic evaluation of limited evidence (195). By changing the expression of the mitochondrial proteins PINK1/Parkin and Bnip3 in osteoblasts, L-arginine increases the activity of vascular endothelial cells and promotes angiogenesis. Moreover, it raises the expression of proteins unique to osteoblasts, including alkaline phosphatase (ALP), RUNX2, and osteopontin (196). Additionally, an intervention that combined L-arginine and allopurinol in osteoblasts decreased the gene expression of pro-inflammatory cytokines IL-6, IL-1β, and TNF-α while increasing the levels of catalase, superoxide dismutase (SOD), glutathione (GSH), and glutathione peroxidase (Gpx) (197). It is crucial to remember that these consequences were not solely attributed to L-arginine. Nonetheless, current findings suggest that L-arginine may positively impact bone injuries in athletes through mechanisms related to anti-inflammation, antioxidant activity, and autophagy. However, clinical evidence supporting these effects remains insufficient.

The body’s most prevalent amino acid is glutamine, whose availability and metabolism are closely related to skeletal muscle tissue (198). Due to its immunomodulatory properties and its role in cellular proliferation, glutamine is widely used in sports nutrition. Research has shown that glutamine supplementation can reduce fatigue by enhancing glycogen synthesis and preventing ammonia accumulation in athletes. By raising IgA and NO concentrations, it also fortifies the immune system, which may enhance sports performance and recuperation (199, 200). Furthermore, L-glutamine reduces p38 MAPK activation in response to TNF-α stress, which is linked to muscle atrophy, according to Girven et al. (201). In addition to preventing muscular atrophy, this activity promotes the development of skeletal muscle cells (201).

It has been demonstrated that using glutamine and arginine together promotes wound healing. A meta-analysis revealed that whereas glutamine supplementation improves nitrogen cycling and lowers concentrations of IL-6, TNF-α, and CRP, arginine supplementation raises hydroxyproline levels (193). Another systematic review found that either arginine alone or a combination of arginine and glutamine supplementation led to a reduction in wound size over a period of 2–20 weeks (202). These results suggest that the studies exhibit significant variation and may only offer indirect evidence for sports-related injuries.

#### 4.4.5 Leucine

Leucine is often regarded as the most anabolic branched-chain amino acid because of its unique ability to stimulate muscle protein synthesis independently. It primarily achieves this by activating the mTOR signaling pathway (203). Leucine plays a crucial role in activating essential molecular pathways, such as AMP-activated protein kinase (AMPK), which promotes muscle protein synthesis and enhances energy metabolism. Additionally, leucine can help prevent inflammation by modulating the NF-κB, MAPK, and Janus kinase/signal transducer and transcription activator (JAK/STAT) pathways, leading to a reduction in the levels of TNF-α and IL-6 (204). In a study of eight male athletes, consuming 2 g of leucine after a meal was found to enhance the anabolic process by influencing specific mTORC1-mediated phosphorylation events (205). Leucine has been reported to reduce muscle damage caused by exercise and is believed to be effective in treating sports injuries (206). In a study conducted by Osmond et al. (207) involving 22 male and female recreational athletes, it was found that the addition of leucine to branched-chain amino acid supplementation did not provide any benefits for post-exercise muscle recovery when compared to standard branched-chain amino acid supplementation. Furthermore, the study concluded that supplementation on its own was not effective (207). When evaluating leucine’s effectiveness in muscle protein synthesis, it’s important to consider dietary factors, including macronutrient and energy intake, training intensity, the timing of leucine intake, and whether it is consumed alongside other amino acids (203). Additionally, the research conducted focuses on the post-exercise period. Thus, further investigation into the role of leucine in sports injuries is necessary.

## 5 Safety issues

Among the primary safety issues in sports injuries are inconsistent supplement contents, a lack of long-term clinical research, uncontrolled use, and individual reaction heterogeneity (22, 109, 123). Concerns continue regarding the misuse of nutritional supplements, including biotics and bioactive compounds, in sports injuries, and the risks of contamination due to a lack of adequate guidance (22). There is currently inadequate data to recommend the routine use of these substances, despite their potential to improve performance and health. It is challenging to elucidate the dose-response relationship and offer a clear recommendation for use due to the small number of studies on polyphenolic supplementation and the inadequate data on the factors (49). These supplements may have negative consequences like gastrointestinal problems and an increased risk of bleeding, especially at higher doses or long-term, according to evidence from systematic reviews (22, 114, 208). On the other hand, labels or manufacturer claims can sometimes be misleading. The type and concentration of supplements for safety concerns must be determined by laboratory analyses in future study (49). To ascertain the safety, effectiveness, and proper usage guidelines of supplements for people with a range of ages and physiological traits, extensive research is required (109).

Gut microbiota-targeted biotic interventions show promise for treating sports injuries; however, caution regarding safety is essential. Although the data currently in publication indicates a high level of tolerance, individual reactions may differ due to the distinct makeup of the gut bacteria. Therefore, the short-term and controlled use of biotics during the post-injury recovery period is crucial to prevent unpredictable changes in the microbiota. Additionally, it is important to consider tolerability when using bioactive compounds for sports-related injuries. For instance, a study on w-3 supplementation in relation to subconcussive head impacts reported gastrointestinal distress among participants (123). Supplementation with amino acids and their derivatives may increase the renal solute load; therefore, it is essential to clearly define the optimal daily dosage and duration of intake (209). Long-term supplementation with L-carnitine has been associated with increased plasma TMAO levels, which may elevate the risk of cardiovascular disease (162). Moreover, dietary intake should also be assessed. Furthermore, the sustainability of these bioactive compounds must be evaluated, as some, like L-carnitine, are derived from animal-based foods.

## 6 Conclusion and future perspectives

Sports injuries are not only a physical problem but also a multifaceted phenomenon affecting an individual’s psychological, social, and professional life. Careful evaluation of the nutrition during the recovery process and its adaptation to varying activity levels is crucial. Because it is stated that nutrition and nutritional supplements (e.g., biotics and bioactive compounds) can be mediators in accelerating healing, reducing the negative effects of immobilization caused by injury, and supporting a return to activity. However, there is a clinical research gap on the effect of biotics and bioactive compounds on sports injuries and muscle mass preservation; definitive recommendations regarding their safe use cannot be made. The available evidence has methodological limitations, including factors such as supplementation duration, dosages used, supplementation forms, and different exercise protocols. Nevertheless, we think that the studied biotics and bioactive substances have potential for treating sports injuries despite all of their drawbacks. Especially, these compounds may serve as adjunct therapies. However, there is a need for standardized dosages of these compounds based on effectiveness testing and clinical studies that have been conducted. The increasing number of subject-specific controlled clinical trials is important for a better understanding of the underlying mechanisms and for the targeted and effective use of biotics and bioactive compounds. Furthermore, the inter-individual variability in responses to these compounds and the factors determining these differences have not yet been established. Key questions for future investigations include: (i) the time-dependent effects of microbiota-derived metabolites and signaling molecules on inflammation and healing after injury; (ii) the efficacy of biotic and bioactive interventions in injured athletes, considering factors like age, training level, nutritional status, and muscle mass; (iii) the impact of combining probiotics, prebiotics, and postbiotics with bioactive compounds on muscle recovery and inflammation control following different types of exercise, as well as the safety of such multi-component approaches.
